# Systematic Review of Noise Pollution in Morocco: Regulatory Frameworks, Urban Impacts, and Policy Recommendations

**DOI:** 10.3390/ijerph23010073

**Published:** 2026-01-04

**Authors:** Mohamed El Malki, Ali Khettabi, Felipe A. P. de Figueiredo, Mohammed Serrar

**Affiliations:** 1 Laboratory of Materials, Waves, Energy and Environment, Department of Physics, Faculty of Sciences, Mohammed First University, Oujda 60000, Morocco; a.khettabi@ump.ac.ma; 2 National Institute of Telecommunications (Inatel), Santa Rita do Sapucaí 37536-001, MG, Brazil; felipe.figueiredo@inatel.br; 3 Faculty of Legal, Economic, and Social Sciences, Mohammed First University, Oujda 60000, Morocco; mohammed.serrar@ump.ac.ma

**Keywords:** noise pollution, noise control policy, regulatory framework, environmental legislation

## Abstract

**Background:** Driven by rapid urbanization, infrastructural development, socio-economic growth, and population increase, noise pollution has become a major public health and environmental policy challenge in Moroccan cities. However, current legislation and enforcement mechanisms remain insufficient to address rising exposure levels and associated health risks. **Methods:** This systematic review followed PRISMA guidelines to examine urban noise levels, health implications, the regulatory frameworks, and policy actions related to noise pollution in Morocco. Various databases were systematically searched (Scopus, Web of Science, Google Scholar), along with reports from international organizations and government bodies for studies published between 2003 and 2025. Eligible documents included peer-reviewed publications and official reports directly addressing Moroccan noise pollution, legislation, urban impacts, or health outcomes. **Results:** Twenty-three Moroccan studies and additional regional, European, and legislative sources were included. Findings show that average noise levels in Moroccan urban centers generally exceed international safety thresholds and are associated with cardiovascular risks, sleep disturbances, and psychological stress. The regulatory framework suffers from weak enforcement, limited monitoring protocols, and an absence of noise mapping. Tangier, Béni Mellal, Témara, Marrakech, and Casablanca exhibit significant environmental inequalities, particularly in low-income districts. **Conclusions:** Morocco’s current noise-management system is inadequate to address the growing health and environmental impacts of urban noise. Urgent actions are needed, including a dedicated noise-control law, systematic monitoring, noise mapping, and integration of public-health considerations into environmental governance. Policy reforms must prioritize vulnerable populations and align with international best practices.

## 1. Introduction

Morocco has been undergoing rapid urbanization. According to the 2024 General Population and Housing Census (RGPH 2024), 62.8% of the Moroccan population lives in urban areas. This represents an increase of 51.4% in 1994 and 60.3% in 2014, demonstrating the rapid shift in the country from a rural setup to an urban one [1], contributing to multiple environmental challenges alongside noise pollution as one of the key public health and policy concerns. These pressures are particularly evident in Morocco’s major cities, including Casablanca, Rabat, Marrakesh, Fez, and Tangier. These metropolitan agglomerations, located along important Atlantic and Mediterranean routes, are crossed by a vast network of highways and port connections between Europe and sub-Saharan Africa, making the environmental issues of transportation a specific concern.

The 2025 National Urban Policy Review of Morocco, provided by the OECD, has highlighted noise pollution as a leading element of environmental nuisances in urban areas, along with air and visual pollution [2]. This has become the case mostly due to heavy congestion in traffic and integrated spatial growth. The United Nations Environmental Performance Review also notes that, although the environmental framework of Morocco has been massively strengthened since the 2000s [3], it still lacks a special and systematic set of noise measurement and mapping that could deliver a systematic evaluation of the exposure level. The urban context, therefore, should include noise management at the heart of sustainable development planning, with an incorporation of the public health, transport, and environment governance agendas.

The World Health Organization (WHO) identified noise pollution as the second most critical environmental health problem after air pollution [4]. The consequences of noise pollution on health are extensive, affecting both physical and psychological health. They include hearing impairment, hypertension, ischemic heart disease, irritation, and sleeping problems [5,6]. Due to their developmental stage and lack of effective coping methods, children are more prone to certain cognitive and physiological effects, while older adults might be mostly affected by cardiovascular impacts, particularly when combined with other factors such as air pollution. However, for annoyance and sleep disturbance, middle-aged adults tend to be more affected than both younger and older groups [7]. Both air and noise pollution from road traffic are recognized risk factors for cardiovascular and respiratory diseases [8].

In Morocco, this issue manifests notably due to its unique geographical position that serves as the link between Europe and Africa, greatly increasing maritime and terrestrial transport activities, which substantially contribute to noise levels in coastal and border cities. The growing populations in cities such as Casablanca, Tangier, Fez, and Marrakech (all with populations exceeding one million) generate increased vehicular traffic, industrial growth, and ongoing construction, increasing noise pollution [9,10]. This level of acoustic stress now rivals that experienced in other large cities, and it is alarming for the long-term quality of life and health of residents, as well as for the effectiveness of existing urban environmental laws.

Furthermore, Morocco’s position in the noise pollution research landscape of Arab countries/regions offers an understanding of what has been achieved while pointing towards considerable remaining challenges. Since the early 2000s, Morocco has made notable progress in terms of environmental legislation, but rapid development driven by infrastructure projects and urban growth has often surpassed the implementation of noise control measures [11,12]. Furthermore, Morocco’s desire to become an economic center for the region presents challenges as it has to manage the demands for economic development, protection of the environment, and issues of public health.

The increase in technology and urbanization presents added challenges for the management of noise pollution in Morocco. The size of the vehicle fleet has increased drastically and is projected to continue growing until 2030. This is projected to be dominated by 80% diesel and 20% gasoline vehicles. This poses a challenge to urban noise pollution, which is already difficult to address with conventional regulatory frameworks [13]. In addition, the patterns of urban development in Morocco have prioritized sprawling, automobile-dependent designs as opposed to compact, transit-oriented developments, further intensifying noise exposure.

The noise pollution in Morocco exhibits the same environmental inequality as that in other developing countries/regions [14]. Low-income neighborhoods are located next to highways, transportation corridors, industrial areas, and construction sites. The inhabitants lack resources for noise pollution alleviation, such as soundproofing or relocation. The negative effects of environmental inequality are compounded by a lack of public understanding of the effects of noise pollution on people’s health and a lack of community participation in environmental decisions [4].

As noted by Benabdallah [15], noise pollution adds to health disparities in Morocco, especially in cities. Families with a low income living near industrial areas and main transport routes face the greatest noise exposures. Such households are often in the peripheral or mixed-use areas, where industrial operations and high traffic volumes converge, causing chronic acoustic stress. The report of the OECD confirms that the accelerated urbanization policy, along with inadequate implementation of environmental policies, has increased the territorial differences in the quality of the environment [2]. The disproportionate exposure to noise and air pollution is observed in the peri-urban and low-income neighborhoods located close to industrial facilities or primary transport routes. These are regions mainly characterized by poor infrastructure, poor insulation, and the absence of social amenities, which make the area prone to environmental irritants and worsen the vulnerability of residents. Moreover, the fact that Morocco does not have a unified national urban policy that addresses the acoustic comfort or noise-conscious urban planning means that these communities remain inadequately protected.

Noise level data obtained from urban areas in Morocco demonstrate significant variations over time and geography, indicating widespread exceedance of internationally accepted safe noise limits during the daytime and nighttime periods. These patterns reflect persistent noise pollution challenges characteristic of rapidly urbanizing cities [16]. The levels of urban traffic noise in Moroccan cities conform in general to international traffic noise frequency-distribution standards, suggesting opportunities to apply international noise mitigation technologies. Survey results indicated that over 45% of respondents are impacted by traffic noise, leading to significant psychosocial effects and widespread sleep disturbance. Reported by 36% of urban residents, it worsens physical and mental health conditions, affects cognition, and impedes socialization. Chronic exposure, especially at night, raises risks for elevated blood pressure, diabetes, weakened immunity, and increased depression. This pollution disproportionately affects vulnerable populations, including children, elderly individuals, disabled individuals, and patients with pre-existing health conditions. Overall, these observations underscore the need for enhanced urban noise monitoring and control measures tailored to the Moroccan context.

According to Adil et al. (2018) [17], predictive noise modeling with CadnaA software using ISO 9613-2 standards was utilized during the first acoustic assessment of Morocco’s first wind farm, Al Koudia Al Baida, inaugurated in 2000. The study highlighted that wind turbines have a significant impact on the surrounding sound environment, particularly at night, emphasizing the absence of specific Moroccan noise regulations for wind energy projects. Given these regulatory gaps, the assessment adopted the German TA-Lärm standards [18] as reference benchmarks for permissible noise levels in mixed-use residential areas. These standards remain internationally recognized and include maximum allowable ambient noise limits for different land-use categories. This approach provided a valuable methodological foundation for assessing the acoustic impact of wind farms in Morocco and supported future identification of high-noise zones around renewable energy installations.

The constant alarms of machinery and sirens at Ibn Sina Hospital in Rabat have long been a subject of discussion, illustrating a notable example of an occupational hazard confronting health professionals and noise-driven stress [19]. A constant stream of distractions impairs one’s ability to think and process emotions, and makes it more difficult to manage stress, exacerbating the psychological toll. Among the study participants, 88% reported low to moderate stress resilience, with 72% identifying noise exposure as the most critical source of stress. Burnout is alarming, with 42% of workers reporting emotional exhaustion, 49% feeling depersonalized internally, and 67% experiencing the least awareness of their professional accomplishments [19]. Additional investigations provide insight into the marked noise-level contrasts, with some areas, such as Ben Kacem, experiencing lower noise levels, whereas others are significantly louder [19]. Noise pollution is a major contributor to psychological distress and burnout among healthcare workers. Therefore, noise control in hospitals should be prioritized for their well-being.

Similarly, the urban growth of Fez is a major contributor to its noise pollution, with nearly 17% of the city already subjected to irritating noise levels, especially in the central areas of the city, which have a concentration of heavy traffic [20,21]. This figure is anticipated to surpass 31% by 2036, serving as evidence of increased use of motorized transport. To address this issue, the Fez Action Plan 2036 proposes a mobility model that aims to reduce the areas threatened by high to medium noise pollution by 45%, targeting the most traffic-congested areas for improvement.

The consequences of noise pollution in Morocco not only include mere discomfort but also cover large-scale exposure patterns that, based on international evidence, can be associated with physiological and psychological health impacts. A significant social survey, developed around the ISO-15666 guidelines, surveyed 1200 randomly chosen adults from Rabat, Salé, and Tetouan. This represents a substantial survey documenting community responses to traffic noise exposure in Morocco, comparable to similar standardized surveys conducted in other MENA countries/regions [22]. The survey revealed concerning trends that expose urban populations to traffic noise. The vast majority of Moroccans’ homes in urban areas are not well insulated against noise. Only 9% of the respondents believed their homes were well insulated against traffic noise. International research focuses on the implications of such environments on stress, cardiovascular function, and cognitive performance. An absence of effective residential noise protection is a strong indicator of inadequacies in building regulations, as well as weaker noise mitigation measures.

In both the short and long term, environmental quality can benefit from financial and industrial development when it is managed sustainably. Urbanization and energy use also influence environmental quality. Policy measures should integrate all environmental concerns, since noise pollution represents only one facet of overall degradation. Urban sustainable-development plans should encompass renewable energy, environmental stewardship, and noise-pollution mitigation. Morocco’s policies on noise pollution are still under development and require improvement to adhere to global norms [4]. Insufficient noise policies in urban low-income areas increase social inequalities and exacerbate noise burdens on underprivileged populations, indicating an urgent need for better policies and the implementation of noise control measures to safeguard these populations.

This work aims to undertake a systematic review of the legislative environment, urban impacts, and health implications of noise pollution in Morocco. The systematic review also compares Moroccan noise standards with international benchmarks to identify gaps in legislation, enforcement, and management strategies, with the goal of informing more effective and sustainable noise-pollution policies for Morocco.

## 2. Materials and Methods

A systematic literature review was performed in this paper, following the PRISMA guidelines [23]. These guidelines assist researchers in replicating a systematic, inclusive, and transparent framework for the processes of study identification, selection, and analysis based on the keywords “noise pollution Morocco,” “urban noise Morocco,” “noise regulations in Morocco,” and “noise pollution health impacts in Morocco.” Boolean operators (AND, OR) were used to put these keywords together in different ways, and adapted them for searches in more than one academic database. Namely: (“noise pollution” OR “urban noise”) AND (“Morocco” OR “Moroccan cities”) AND (“health impact” OR “public health” OR “noise regulations”).

**Figure 1:**
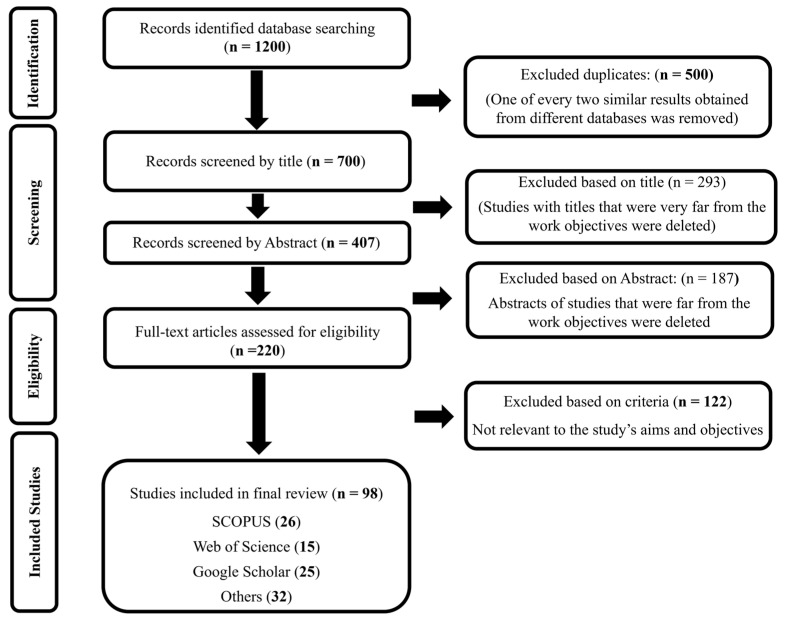
PRISMA 2020 Flow diagram for study selection.

In this case, the objective is to evaluate the regulatory frameworks, urban implications, and policy implications associated with noise pollution in Morocco (Figure 1). The systematic review was initially designed to conduct a preliminary search covering the years 1990 to 2025 comprehensively to guarantee proper coverage of all possibly relevant literature. Nevertheless, the decision on the final study period was made according to the a priori criteria that had been set during the protocol development, such as:

- Legislative Milestone: The year 2003 marks the enactment of Morocco’s Law related to noise pollution. This law is considered the first comprehensive environmental protection legislation of the country, dealing specifically with noise pollution. It is with the passing of this law that Morocco sees the mapping of the modern era of noise pollution governance and the creation of the regulatory context that this review is concerned with.
- Evidence Availability: Initial scoping searches that were done across scientific databases for the years 1990–2002 did not yield any peer-reviewed studies or institutional reports that had noise pollution in Morocco as their subject. The non-existence of any systematic noise study before 2003 is indicative of the absence of official frameworks and institutions capable of carrying out noise monitoring during the earlier time.
- Relevance to current policy context: The literature after the passing of the noise pollution Law can be considered equivalent to current and future policy issues and thus can serve for the evaluation of regulatory implementation, the identification of enforcement gaps, and the making of evidence-based policy recommendations within Morocco’s existing legal framework.

The search was conducted from January 10 to 15 August 2025 (publications in English, French, and Arabic only). Specifically: identification/screening (15 January–April 2025), eligibility (full-text, May–June 2025), included studies (extraction/synthesis, July–15 August 2025), using the full Boolean strings and 2003–2025 inclusion criteria. Besides a database search, systematic searches of the grey literature were also carried out, such as reports written by UNECE, WHO, and national environmental agencies (see the Supplementary Materials). The registration of all search strategies was performed and replicated by two independent reviewers to provide reproducibility and reduction in search bias.

The studies involved both quantitative and qualitative research, with cross-referencing of noise pollution legislation and research that meets international standards, namely those of the European Union and some Arab countries/regions.

The pertinent studies were first retrieved via carrying out exhaustive searches of a series of databases: Scopus, Web of Science, and Google Scholar. The keywords searched were: noise pollution in Morocco, urban noise in Morocco, noise regulations in Morocco, and noise pollution health impacts in Morocco. Furthermore, reports from other official sources, including UNECE, WHO, and local environmental agencies (such as RGPH), were also included to incorporate relevant literature.

Systematic retrieval of the relevant studies was carried out in terms of gaining an exhaustive search in four of the largest bibliographic databases, namely Scopus, Web of Science, and Google Scholar. Each database received a standardized search strategy that was tailored to the database, with the addition of Boolean operators and controlled vocabulary where necessary. The searching formula, which was used in all databases, was the following:

- Scopus (Elsevier): TITLE-ABS-KEY ((“noise pollution” OR “urban noise”) AND (“Morocco” OR “Moroccan cities”) AND (“public health” OR “health impact” OR “noise regulations”))
- Web of Science Core Collection: ((“noise pollution” OR “urban noise”) AND (“Morocco” OR “Moroccan cities”) AND (“public health” OR “health impact” OR “noise regulations”))
- Google Scholar: (“noise pollution” OR “urban noise”) AND (“Morocco” OR “Moroccan cities”) AND (“public health” OR “health impact” OR “noise regulations”).

The comprehensive data search via databases led to the identification of 1200 records in the first instance (Figure 1). With the elimination of 500 duplicates, there were still 700 records left for title screening, out of which 293 were rejected because the titles were evidently unrelated to the review objectives. The 407 records that were left were screened by their abstracts, and 187 were ruled out at this point for not corresponding to the study’s area. A total of 220 full-text articles were then evaluated for their eligibility, and 122 of them were rejected according to the already set inclusion and exclusion criteria (not pertinent to the study’s aims and objectives). Finally, 98 studies were incorporated in the qualitative synthesis and analysis (Figure 1). Namely, 26 publications from Scopus, 15 from Web of Science, 25 from Google Scholar, and 32 documents from other online platforms.

The following criteria were considered for the studies:

- Papers related directly to noise pollution subjects in Morocco or comparative studies involving Morocco.
- Studied either of the following: regulatory frameworks, urban noise levels, or health impacts.
- Published in peer-reviewed journals and institutional reports.

The exclusion criteria included the following:

- Studies not relevant to noise pollution or those that discuss other forms of environmental pollution
- Studies without empirical data or that do not provide an understanding of the policy and urban components of noise pollution.

Data extracted from the relevant studies included the following:

- Study aims and outcomes (e.g., health impacts, regulatory frameworks, and urban noise levels).
- Methods of measuring noise (e.g., L90, Leq, and day/night noise distinctions).
- Comparative data on noise levels in Moroccan cities compared with international norms.
- Gaps in the legislative framework and policy recommendations.

Two independent reviewers used a standard data extraction form. The form incorporated the study objectives, methods for noise measurements, results, and policy implications. The extracted data underwent a second check by a third reviewer to isolate errors and ensure their correctness. 

Assuming that all the tasks above were performed, a comparative analysis was conducted between the noise pollution situation in Morocco and international best practices, particularly focusing on the European Union’s noise directives and noise management policies in Arab countries/regions. The comparison highlights the differences in the sophistication of the regulations, enforcement mechanisms, and public health approaches.

### Characteristics of the Included Studies

Thereupon, the first arm ensued in the systematic identification of studies on noise pollution in Morocco, applying PRISMA for cleaning and screening literature (n = 23), while the second arm consisted of targeted evidence collection combining Arab regional studies and legal frameworks (n = 48), EU legislative frameworks (n = 13), Moroccan legal documents (n = 6), international standards (n = 6), and methodological references (n = 2) for allowing comprehensive comparative analysis with policy recommendations. The data were then synthesized under urban impacts, regulatory shortcomings, health effects, and recommendations to improve policies.

**Figure 2:**
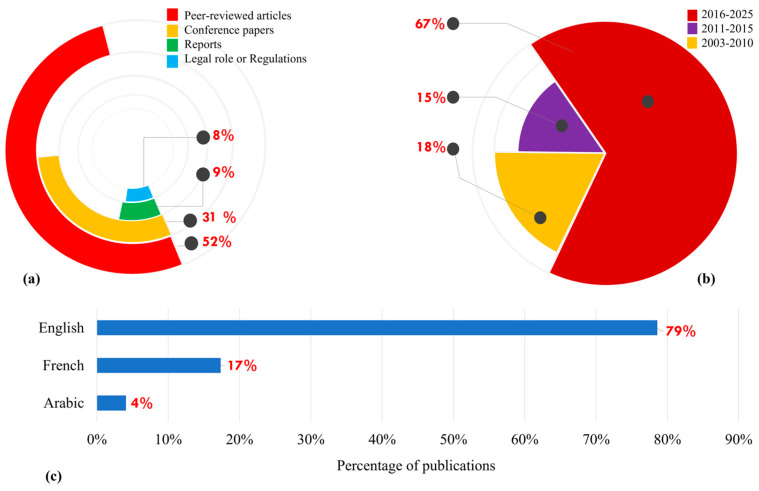
Distribution of the included publications by (**a**) type, (**b**) publication period, and (**c**) language.

To enhance transparency, the 98 records that were all screened on a full-text basis were described in terms of the document type, date of publication, and language distribution (Figure 2). Peer-reviewed journal articles accounted for 52% of the corpus (Figure 2a), while conference papers represented 30%, reports 9%, and legal or regulatory texts 8%. This spread explains that the journal publications prevail, but a substantial share of the evidence base still derives from grey literature and conference proceedings. The majority of included peer-reviewed articles were retrieved from Scopus and Web of Science, while conference papers, reports, and legal documents were mainly obtained through Google Scholar, institutional repositories, and official government portals. Because the initial search logs did not systematically record the exact proportion of records per individual database, only the combined total from all databases can be reported.

The distribution of the number of publications over time indicates a distinct increase in the activity of the research. When divided by period (Figure 2b), 18% of the total studies were published within the year 2003–2010, 15% in the year 2011–2015, and 67% in the year 2016–2025, which shows that there has been a rapid increase in the interest of noise pollution and other environmental effects in recent years. Even though, among the 22 countries/regions in the League of Arab States, Morocco is ranked at the lower end of research productivity in studies about noise pollution for the period 1983–2022, compared with countries/regions such as Jordan (17 studies), Saudi Arabia (16 studies), and Kuwait (15 studies) [24]. This reduced research output, constituting just 1.9% of total publications from the Arab world on noise pollution, highlights a substantial expertise and evidence gap that undermines policy development through an evidence-based approach within the country [4,24]. However, research output in this area has accelerated across the Arab League, including Morocco, in recent years [25,26,27,28,29,30,31,32,33]. These recent studies aim to bridge the existing research gap and revitalize noise pollution-related policy development, thus holding greater promise for stronger legislative action and decision-making.

In terms of language (Figure 2c), 79% of the included documents were published in English, 17% in French, and 4% in Arabic. Although no restrictive language was applied to the review, the Arabic publications are mostly devoted to presenting or analyzing international and regional noise-control regulations, whereas the documents in the French language are composed mostly of legal texts and official national or ministerial reports on the environmental and noise regulations. This trend can be understood as the prevalence of English in the scientific literature as well as the propensity towards writing regulatory and institutional material in Morocco in French or Arabic with regard to the effects on the ability of various audiences to access and use the evidence base. All included publications are available online, either through open-access platforms, institutional repositories, or official governmental portals.

In addition, thematic and institutional analysis has shown that the majority of the Moroccan empirical works stem from a small group of academic and especially physics, engineering, environmental science, and public health laboratories of national universities and related institutes. Yet, such concentration suggests that the field is driven by a limited set of specialized teams rather than a broad national network, underlining the need to expand institutional engagement.

## 3. Analysis of Noise Pollution Perception Across Moroccan Cities

In this section, we synthesize city-level evidence on noise pollution in Morocco, combining objective measurements, case studies, and perception-based indicators.

Increasing traffic congestion has become a concern in Casablanca, Morocco’s largest city, particularly along the Ain Harrouda to Lissassfa highway. The city is also experiencing rapid urban development, which is a contributing factor to rising traffic, and Casablanca’s contribution to noise pollution is important. According to a previous study that also assessed the impacts of a bituminous coating and an extra lane, the improvements in noise levels reached 23 dB (A) during the peak hours from 10 AM to 4 PM [34]. Through construction-based alteration in many cities, changes in road layout due to construction work actually reduce traffic and hence noise pollution. In contrast to Casablanca’s industrial and traffic-related challenges, other Moroccan cities face different sources of urban noise linked to their unique socio-economic activities.

Tourism’s rapid global expansion has intensified urban noise pollution challenges worldwide, particularly in popular destinations. In Morocco, this manifests as over-tourism pressures in historic cities. Marrakech, a UNESCO (United Nations Educational, Scientific, and Cultural Organization) World Heritage Site and major tourist hub, exemplifies this issue. From 2017 to 2019, Marrakech was ranked as the 6th-worst city in the world in terms of sleep deprivation [35]. Reflecting the high noise levels, approximately 60% of the hotels reported that guests struggled to get a restful night’s sleep [35]. This continuous noise, together with heavy tourist traffic and crowded markets, harms the well-being of tourists and locals alike. Marrakech’s tourism-driven noise pollution persists despite general urban planning challenges, particularly urban sprawl pressures in historic areas [2].

Industrial development in eastern Morocco (population: 2.26 million across 90,127 km^2^) contributes to complex noise control challenges, particularly from manufacturing and transport activities lacking systematic acoustic regulation [36]. These industrial noise issues reflect a broader pattern of environmental noise pollution that could affect diverse areas in Morocco. 

A study conducted at Sidi Yahya Zaer Middle School in Témara demonstrated that sound levels during class exceeded accepted norms and hence adversely affected students’ ability to concentrate and learn, teacher performance, and the overall educational environment [10]. This study underscores the need for noise-control strategies in schools, such as the acoustic isolation of school buildings and stringent zoning. To address these issues, Morocco must increase public awareness of noise pollution and its health impacts, integrating urban health principles into development models to improve living conditions and health [37,38,39]. In addition, a study spanning from 1971 to 2019 [33] examined, using the autoregressive distributed lag (ARDL) method, the influence of CO_2_ and environmental quality on per capita income, energy use, urbanization, and value-added manufacturing, as well as the reverse. Research conducted by Ammar et al. [40] on students living in urban areas of Morocco revealed that students, especially those living in disadvantaged neighborhoods close to major roadways, suffer from an unbearable level of road traffic noise daily.

**Figure 3:**
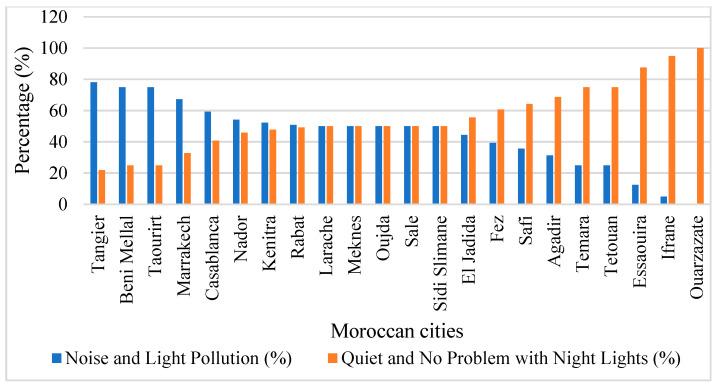
Citizen perception of noise and light pollution across Moroccan cities.

Figure 3 gives a comparative analysis of the environmental quality perception in 20 cities in Morocco. Two major indicators were the noise and lighting pollution (depicted by the blue color) and the quietness of the surrounding environment with no trouble with night lights (depicted by the orange color). These data were sourced in Numbeo [41], the largest crowd-sourced list of perceived quality-of-life measures in the world that gathers data by a mixture of user-generated questionnaires and institutional data collected by user-verified sources using a combination of both. The methodology applied in Numbeo survey on pollution index survey uses a structured survey approach whose respondents are asked to rate their perception of the environmental condition using the standardized scale ranging between the lowest index (very low −2) and the highest index (very high +2) across several aspects of pollution, such as air quality, noise and light pollution, water quality and cleanliness. The platform uses more than 30 advanced algorithmic filters to guarantee the accuracy of data and remove spam entries, and data generated on government and institutional levels are rated with three times the weight of user-generated inputs to make the platform more reliable.

More specifically, for noise and light pollution, respondents are asked to report their subjective experience of acoustic nuisance and light in their homes, the responses are aggregated into the percentage scales which reflect the share of the residents who report feeling the problem (noise and light pollution) against the share of residents who report to have a satisfactory experience (quiet and no problem with night lights). This perception-based approach provides important insights into how residents experience environmental quality, particularly where objective measurement data are unavailable.

The data also depict a remarkable geographic and developmental gap in the extent of environmental quality perception, with major urban centers reporting the highest levels of noise and light pollution, and smaller cities and urban peripheries displaying negative environmental quality curves. The highest levels of noise and light pollution are recorded in major urban centers, including Tangier, Béni Mellal, Témara, Marrakech, and Casablanca, which are also the cities with the lowest levels of satisfaction with a quiet environment and darkness. The trend is indicative of the economic activity and traffic concentration, the density of the activity, as well as the nighttime commercial activity of major metropolitan areas in Morocco, where over 60% of the population now resides. Tangier has 78% of its population indicating the presence of noise and light pollution, with 22% of the population reporting poor living conditions with respect to noise and light pollution, which is the worst ratio among cities that were surveyed, given that it is also a major port in the Mediterranean and an industrial center with 24-h activities producing constant noise and lighting. Casablanca (60% perception of pollution, 40% satisfaction) and Marrakech (67% pollution perception, 33% satisfaction) experience the same pressures of traffic congestion, tourism infrastructure, and urban dense development. Beni Mellal (76% pollution perception) and Témara (75% pollution perception) are urban growth areas with high and fast population growth and infrastructure development, where residential areas are becoming more and more exposed to industrial and commercial estates without sufficient spatial buffer and environmental regulation.

In stark contrast, smaller cities and peripheral urban areas, especially Ouarzazate, Ifrane, Essaouira, Tetouan, and Témara, have better environmental quality. Ouarzazate achieves an almost 100% of locals who testify to quiet conditions, with Ifrane (95%), Essaouira (88%), and Tetouan (75%) also portraying high satisfaction percentages. These results reflect less activity in industries, and the geographical location is remote from important transport routes. Ouarzazate’s extraordinary performance reflects its desert location with minimal urban sprawl, and low population density.

The profile of mid-spectrum urban centers, such as Kenitra, Rabat, Larache, Meknes, Oujda, Sale, and Sidi Slimane, exhibits a balanced view of the perceived pollution, with 48–55% reporting pollution problems and 45–52% reporting satisfactory conditions, indicating the presence of transitional pressures related to urbanization but with the remaining quality environment.

### Traffic Congestion in Morocco: A Comparative Analysis

Because road traffic constitutes the predominant source of urban noise exposure in Moroccan cities, it is important to place city-level noise perceptions in the broader context of national and regional traffic congestion trends. Traffic congestion is an essential measure that facilitates the diagnosis of traffic issues in cities, empowers infrastructure sustainability, and determines the quality of life in fast-urbanizing communities. The traffic index, which is a metric that measures congestion by the use of commute time, systemic travel time, and system inefficiencies, provides a quantifiable rubric for assessing the performance of urban mobility within various contexts of metropolitan areas. In Morocco, a subtle perception of the local traffic congestion in the country, on both local and global platforms, is invaluable in putting in place evidence-based structures in the framework of infrastructure planning and development.

In this respect, the resulting comparative analysis on the performance of traffic in Morocco puts this perspective in the context of the Arab League member states and international standards, thus shedding light on the current issues and adding insights on the future paths of approving sustainable urban mobility interventions.

**Figure 4:**
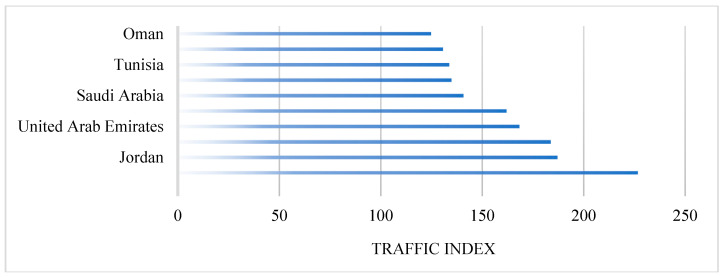
Comparative traffic index across Arab countries/regions [42].

Traffic congestion in the Arab world shows that Morocco has an average traffic congestion compared to the other countries/regions of the region. As shown in Figure 4, the traffic index (TI) in Morocco is 130.7. The index testifies that the Moroccan performance is the best in comparison with some of the most overcrowded Arab nations, including Egypt (TI = 226.7), Jordan (TI = 187.1), Lebanon (TI = 183.8), the United Arab Emirates (TI = 168.4), and Kuwait (TI = 162). Morocco’s traffic index is close to that of Tunisia (TI = 133.9). However, it is better than Qatar (TI = 134.9), and Saudi Arabia (TI = 140.9), even though those countries/regions have significantly higher GDP per capita and investment in infrastructure. However, Morocco experiences notably higher congestion than Gulf states with superior infrastructure, particularly Oman (TI = 124.9).

**Figure 5:**
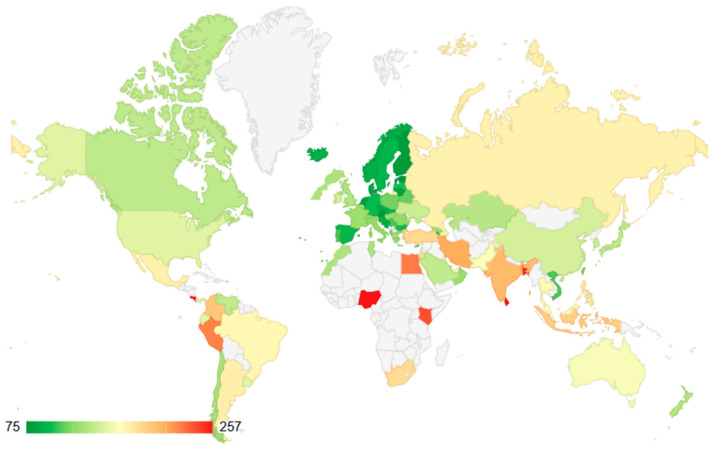
Comparative traffic index across the world [42].

Globally, Morocco is ranked 51st among the countries/regions measured in the 2025 mid-year traffic index, thus being classified in the section of international traffic in the upper-middle segment of traffic congestion. Figure 5 provides a more international picture in that Morocco is presented as a country among about 50 countries/regions with traffic indices below 100 (high efficiency) and above 300 (high congestion). The traffic index for Morocco (130.7) is close to but slightly higher than the values reported for Greece (127.9), Georgia (127.4), and France (126.5), indicating that Morocco experiences congestion levels comparable to or somewhat worse than many higher-income European countries/regions. This seemingly contradictory situation reflects shortcomings in road and public-transport infrastructure capacity relative to the pace of rapid urbanization, rather than an exceptionally high absolute vehicle density. Morocco’s position in global congestion rankings suggests that its traffic conditions resemble those of middle-income countries/regions undergoing fast urban growth, where road network development lags behind the concentration of population and economic activity in large urban centers.

## 4. Legal Framework: Assessment of Morocco’s Noise Control Legislation

Law No. 11-03 is Morocco’s principal legislation for governing noise pollution. It was enacted following Dahir No. 1-03-59 on 12 May 2003 to establish environmental protection and enhancement objectives [43], and its implementation is supplemented by a Decree 93-08 issued on 12 May 2008 [44]. This environmental law provision provides the fundamental legal framework and support for protecting the environment in Morocco, and includes provisions dealing with noise pollution that are the first systematic attempt at enacting national legislation aimed at acoustic pollution.

As a comprehensive environmental law, this mandate offers both opportunities and challenges for implementing noise control. The law’s definition of environmental pollution is “any direct or indirect influence on or modification of the environment that arises from human acts or activities or that arises from natural factors. Any one of these impacts or alterations to the environment may cause damage to public health, safety, or well-being, or pose a danger to the natural environment, property, values, and legally sanctioned uses of the environment”. This all-encompassing definition of noise pollution is situated within the broader framework of environmental protection [43]. While these expansive definitions reflect international best practices in environmental law, they hinder implementation, largely owing to concerns about allocating limited enforcement resources and prioritizing compliance efforts.

Law No. 11-03, Article 3 defines polluting substances and factors in a specific manner in which it is defined as all solid, liquid, or gas materials, or noise or radiation or heat or sound vibration produced by human activity that has directly or indirectly caused environmental pollution or degradation. Building upon this definitional framework, administrative responsibility becomes the realm of an administration under Article 7 of the same Law, whereby concerned administrations shall take all necessary measures to protect settlements from negative effects resulting from any form of pollution and disturbance from solid waste and liquid or gaseous discharges as well as all forms of noise and vibrations that do not comply with environmental quality standards and criteria.

The substantive regulatory provisions are detailed in Article 47 of Law No. 11-03 [43], which addresses noise pollution. The article states, “Noise and sound vibrations of whatsoever type and source shall be reduced or eliminated if likely to cause a disturbance to neighbors or on account of human health or the environment in general, particularly during production and servicing activities, operation of machinery, equipment, warning devices, and loudspeakers.” The article also states that the regulatory texts shall determine “the maximum permissible limits for sound levels and the cases and conditions in which the emission of any noise or sound vibrations shall be prohibited, as well as the measurement methods and monitoring techniques.” However, no specific technical details are provided. In this way, Law 11-03 formally recognizes the necessity of precise measurement and monitoring procedures while entrusting their detailed definitions to subsequent decrees and ministerial orders.

The Decree 93-08 of 12 May 2008 mandates systematic noise monitoring and initial measurements wherever exposure meets or exceeds the 85 dB(A) continuous or 135 dB(C) peak, requires re-assessments every three years or after any change likely to increase noise, obliges employers to reorganize work or implement noise-reduction programs and to provide hearing protectors that ensuring residual exposure stays below those thresholds, permits only medically certified employees to perform high-noise tasks under regular audiometric surveillance, and compels comprehensive worker information and training on noise risks, prevention, protective equipment, and medical follow-up.”

In addition to the national environmental statutes, Moroccan communal law and joint-ownership regulations impose direct noise controls at the property level. Under Decree No. 2-17-354 of 3 October 2017 (Model Regulations for Joint Ownership), Article 17 explicitly forbids any co-owner or their substitute from creating or permitting noise that disturbs the tranquility of the jointly owned property, equipment liable to produce noise must be used in moderation so that no sound is heard beyond the premises, and the owners’ association representative may suspend any work causing noise, distinguishing between ordinary working days and holidays [45]. Law 113-14 of 7 July 2015, Article 100 empowers the President of the Communal or District Council to exercise administrative-police powers over health prevention, public hygiene, public tranquility, and traffic safety through regulatory decisions and individual measures (permissions, orders, prohibitions) to prevent noise and environmental pollution [46]. Article 236 of the same law further provides that these administrative-police powers may be delegated, in whole or in part, by the Council President to deputies or senior communal officials under his responsibility and subject to identical controls, including measures to combat noise and other nuisances [46].

Furthermore, Law No. 12-03 on Environmental Impact Studies, promulgated by Dahir No. 1-03-60 on 12 May 2003 [47], was Morocco’s first environmental impact assessment legislation until it was repealed by Law No. 49-17 in 2020 [48]. The law concerning environmental impact studies laid down the framework for requiring an environmental impact study for any given project, infrastructure development, or industrial activity. Although it does not address noise pollution limits, noise is considered insofar as environmental impacts on public health, hygiene, and safety are concerned, in addition to the discharge of liquid waste, gaseous emissions, and solid discharges. The law also provided for public hearings for local communities to convey their concerns and had enforcement options whereby the relevant authorities could order the suspension of ongoing projects for noncompliance. Subsequently, Law No. 49-17, enacted under Dahir No. 1-20-78 in 2020 [48], replaced Law No. 12-03, which focuses on environmental assessment. It theorizes the existence of “acoustic nuisances, luminous and olfactory disturbances, and damage caused by heat and radiation” during the implementation and operation of projects. Project proponents are, thus, obliged to assess direct and indirect environmental effects, including noise, and set up programs for monitoring and follow-up to ascertain compliance with environmental standards.

The advent of comprehensive noise measurement and health-impact assessment methods has augmented the capacity for environmental health research in Morocco. Continuous monitoring of noise at a height of 1.5 m and a distance of 2 m from building facades provides internationally comparable data in accordance with the NFS 31-110 standards and reflects Moroccan building designs and urban characteristics [16]. This level of detail in methodology enables a detailed assessment of noise-exposure patterns, on the basis of exposure classifications and specific health outcomes.

### 4.1. Analysis of Morocco’s Legal Framework for Noise Pollution: Regional and International Context

The development of noise pollution regulations in the Arab world began in 1966 in Iraq, which enacted an important law, intended to limit the use of loudspeakers in public spaces. This law comprised nine articles and was the first response to concerns surrounding environmental noise, particularly in densely populated cities such as Baghdad and Cairo, where daily urban life features high noise levels. This growing concern prompted many Arab governments to address the issue. It introduced penalties, fines, and imprisonment for noise from amplified sounds that disturbed public peace, and subsequently set a legislated framework for other legislative actions to take place.

In the 1980s, other Arab countries/regions, including Algeria and Tunisia, followed Iraq by embedding noise control within broader environmental and urban-development policies [49,50,51,52]. These early regulations paved the way for more systematic noise legislation across the region. The 1990s saw a notable growth in noise legislation, reaching several countries/regions, including Egypt, Oman, Kuwait, Yemen, Bahrain, the United Arab Emirates (UAE), Lebanon, Palestine, Saudi Arabia, and Syria, which developed regulations related to noise pollution [53,54,55,56,57,58,59,60,61,62]. For example, Saudi Arabia’s 2001 national noise policy was substantially revised in 2019 [63].

Similarly, Kuwait showed that it was no longer building its environmental protection framework from scratch, developing an environmental protection law in 2014 [64]. A comprehensive overview of noise-pollution legislation in selected Arab territories is presented in Table 1, with additional laws detailed in [4,63,65,66,67,68,69,70,71,72,73,74]. Environmental noise laws always have this feature: they are incorporated into broader environmental- or air-pollution statutes rather than enacted as standalone legislation. Noise in countries/regions such as Kuwait, Lebanon, Libya, Mauritania, Morocco, and Qatar is usually considered a general environmental nuisance and is rarely seen as a distinct public health issue. This approach, while a step forward, sometimes leads to enforcement difficulties and legal ambiguities.

Distinct features of noise pollution laws in the Arab world include their integration into broader environmental protection frameworks or air pollution legislation, rather than being established as independent laws [88]. In countries/regions such as Kuwait, Lebanon, Libya, Mauritania, Morocco, and Qatar, noise is often treated as one aspect of broader environmental concerns rather than as a separate public health issue. While this approach marks progress, it can sometimes result in enforcement challenges and legal ambiguities.

In contrast, countries/regions such as Oman, Algeria, Iraq, and the Kurdistan Region of Iraq have developed national noise and vibration control regulations that are separate from general environmental pollution laws, and are enforced by environmental or municipal bodies independent of the central government. These measures address specific noise- and vibration-nuisance issues directly. Similarly, municipal or regional regulations manage such disturbances in Tunis, Somaliland, and Kurdistan, independent of national legislation.

The regulation of noise pollution in Morocco reflects a broader challenge across the Arab world, characterized by diverse legislative frameworks and enforcement approaches. Almost all Arab states, including Morocco, have enacted noise legislation. However, these laws seldom specify technical details for measuring or mapping noise levels. This absence of detailed guidance has created confusion in municipal enforcement. Unlike some Arab countries/regions in the region, Morocco treats noise under the general scope of environmental laws, all of which face difficulties in implementation and enforcement. It is slightly upgraded, but vague, noisy environmental laws enhance diverging interpretations. Thus, it creates confusion in enforcement and eventually renders the laws impractical.

Countries/regions such as Saudi Arabia, Oman, and Qatar have issued specific noise-and-vibration decrees enforced by local authorities rather than national ministries, enabling a more systematic approach. Nonetheless, even these decrees often lack precision; many regional laws remain too vague to enforce effectively. As seen in Morocco, Lebanon, and Mauritania, the lack of technical detail renders enforcement impractical. Thus, Morocco should adopt comparable noise-policy measures aligned with international standards, adapted to its sociocultural and urban contexts.

The recommended measures include establishing clear governance of noise thresholds, conducting noise-mapping exercises, and increasing public awareness and engagement in noise control initiatives. This would help Morocco shape its laws based on examples from countries/regions where noise has been given representation through detailed, enforceable regulations to protect public health and the environment from the effects of noise pollution. A notable gap exists in regional environmental-health safeguards addressing public exposure to vibrations. Noise pollution is often regulated, but there has been little legislation seeking to address the health effects of exposure to vibrations in the Arab world. There are a small handful of Arab countries/regions (e.g., Oman, Saudi Arabia, and the UAE) that have considered health-related vibration controls as part of their noise decrees, addressing public exposure and health effects from vibrations such as physiological stress or annoyance, although few states have comprehensive vibration-specific laws or mapping schemes, providing an opportunity to progress [88].

Several nations in the Arab world have comprehensive, enforceable noise laws. Saudi Arabia, Egypt, Oman, the UAE, and Qatar have structured legal frameworks that set out permissible noise levels, zoning regimes, monitoring systems, and often environmental impact assessments (Table 2). These countries/regions are among the few in the region that differentiate between daytime and nighttime noise levels and specify permissible noise levels in residential, industrial, and sensitive zones. These structured legal frameworks are examples for other countries/regions in the region to build upon. Even though all noise laws in the Arab world are enforceable, enforcement varies significantly. Many still lack the necessary technical standards, noise-mapping capabilities, and public health protocols.

Only a small number of countries/regions (i.e., Egypt, the UAE, and Saudi Arabia) [88] have established noise monitoring stations and aligned their noise assessment procedures with international standards (e.g., ISO 1996-1:2016). Approximately 11 out of the 22 Arab League countries/regions have enacted standalone noise laws rather than embedding them within general environmental statutes. At the same time, at least 10 Arab countries/regions have noise legislation, but few have passed legislation. Although ten Arab states now have specific noise legislation, Morocco’s Law 11-03 lacks the technical implementation mechanisms found in more advanced frameworks such as those of Saudi Arabia, Qatar, and Oman, which include measurement protocols, technical guidelines, and day-/night noise distinctions [88]. No Arab state has yet established comprehensive noise-mapping systems [4], highlighting a critical regional gap that Morocco could pioneer by developing its own mapping system.

The countries/regions separated by their degree of noise policy framework development are leaders, evolving systems, and others needing improvement (Table 3). Morocco is placed under category number three among Arab countries/regions, as there is no legislation or the legislation is empty, with serious gaps in the area of technical application. Morocco is better than 13 Arab countries/regions that have no specific noise legislation, but it falls behind regional leaders such as Saudi Arabia, Qatar, and Oman in establishing rigorous permissible limits and technical guidelines.

Consequently, although the Arab world has made fairly impressive progress towards controlling environmental noise, there remain issues with enforcement, legality, and response to vibrations, which are rising issues. Regional legal frameworks are evolving, and we view this as a process (often slow), moving towards a better-guided process, and many gaps remain, especially as they apply to coherence on a national basis and to enforcement processes.

### 4.2. Comparative Analysis of Noise Pollution Legislation: Morocco, the European Union and the Arab World

A systematic comparison between Moroccan survey findings and those obtained from similar surveys in several European cities identified differences in both the levels of noise exposure and the levels of adaptive response by the community [4]. The comparative analysis reveals not only the magnitude of noise pollution in Moroccan cities but also the necessity for Morocco to create a locally based noise control policy and measures against contemporary standards while accommodating the country’s urban conditions and cultural context. When analyzed alongside the European Union Standards, the deficiencies in Morocco’s dynamic noise policy (Table 4) are evident. Noise pollution in many EU cities is usually best managed under a strong regulatory framework [89,90,91,92,93,94,95,96,97], with integrated urban planning, strict standards on noise generated by vehicles, and wide-ranging noise-mapping schemes. Moroccans endure noise levels that could be considered unacceptable in the European context, yet they continue to find themselves with fewer resources and safeguards to address noise exposure. This disparity again emphasizes the need for international cooperation and technological transfer towards engineering, which is a realistic noise-control mechanism for Morocco.

While Morocco has a legal framework addressing environmental pollution (Law No. 11-03), the country has yet to establish detailed and structured noise management programs akin to those in the EU. The European Noise Directive stands as a highly complete model for noise pollution management, setting noise limits for different zones, requiring noise mapping, and promoting ongoing noise measurement. This framework allows most EU countries/regions to reduce noise pollution in urban areas, improve the health of their citizens, and provide a better standard of living. Morocco would gain much if it adapted some of the noise management approaches of the EU in consideration of its socioeconomic and urban profiles.

The expression of environmental noise is another major consideration within Arab countries/regions as well as the European Union. Nevertheless, the legislative framework greatly varies in structure, scope, and enforcement. Most Arab countries/regions have adopted some type of legislative framework to regulate noise pollution, but this legislation is typically fragmented and much less extensive and enforceable than the frameworks developed in the EU. In the Arab League, several countries/regions have successfully incorporated noise regulations into their broader environmental legislation, whereas only a few countries/regions (e.g., Algeria, Egypt, and Saudi Arabia) have developed specific laws to address noise. Morocco enacted a law regarding pollution abatement. Yet, it does not set out any rigorous permissible noise limits, which undermines the overall viability of the law (Table 5). In contrast, the EU was able to establish a more structured and detailed framework for environmental noise management, including specific directions under Directive 2002/49/EC, which requires member states to assess, manage, and reduce ambient noise exposure. This directive outlines the requirements for mapping noise exposure, setting permissible noise limits, and developing noise action plans for urban areas, providing some assurance that noise as a pollutant is being planned for and managed throughout the lifetime of the community.

Thus, while Morocco has established foundational noise control laws through Law No. 11-03, there are significant gaps that remain in its implementation. For example, there are no specific permissible noise limits stipulated in the legislation, and no noise monitoring capacity, with negligible enforcement capabilities.

Concerning further developed frameworks, for example, the European Union and Directive 2002/49/EC, whereby the conditions set out are a clearly defined level of noise for different types of zones (residential, commercial, industrial), noise mapping, and regular monitoring of ambient noise levels, Moroccan laws are less detailed. The absence of these technical elements, or similar tools such as limits on the level of noise, how to estimate noise levels, measurement methodologies, and noise or acoustic monitoring, makes it impossible to assess and manage noise pollution properly throughout urban areas, where the impact is usually greatest. Another key difference is the standards for permissible noise levels. Some Arab countries/regions, including Egypt and Jordan, have established noise limits, but these regulations are often not detailed. In many cases, they fail to distinguish between different areas, such as residential and industrial zones, where noise sensitivity varies. This lack of specificity makes the enforcement of noise regulations more difficult.

Morocco is an example of this, since it does not determine strict limits or designate particular noise zones, limiting its application. European Union laws are the opposite. The EU has predetermined noise limits for each noise zone as well as for specified noise sources for noise produced by vehicles and from industrial sources, which are regulated by Regulation No. 540/2014 (EU) for motor vehicle noise, and Directive 2010/75/EU for industrial noise emissions. The legislation also requires establishing permissible levels, ongoing monitoring requirements, and strategic noise mapping for member states of the EU that will support enforcement and planning.

**Figure 6:**
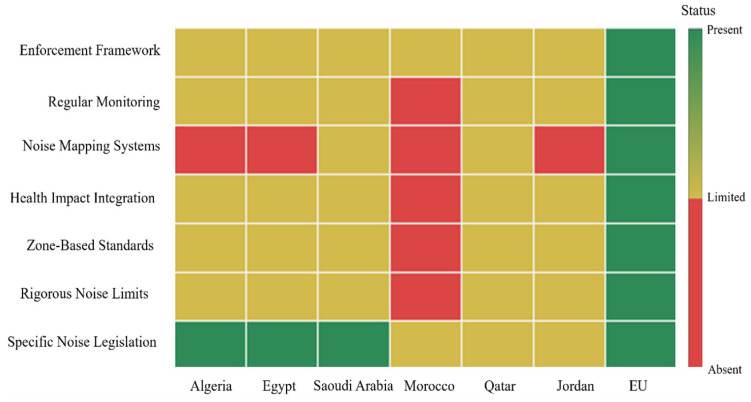
Development status matrix of noise regulation components in Arab countries/regions in comparison to the EU.

The discussion of the health and environmental effects of noise differs immensely between Arab countries/regions and the EU. Arab countries/regions clearly focus on the nuisance and quality of life issues concerning noise, whereas the EU incorporates public health issues into its legislation (Figure 6). In the EU, noise pollution is linked to public health issues related to cardiovascular diseases, sleep disorders, and mental health problems, and these concerns are addressed in noise regulations. In contrast, Arab countries/regions such as Morocco have not yet fully incorporated the health impacts from exposure to noise into their laws, and only a few studies have been published on the health impacts of environmental noise. Nevertheless, the legislation in the EU makes an effort to accommodate noise control for public health and well-being purposes and incorporates scientific research into all laws and regulations to minimize exposure to noise intrusions in areas with a high population density.

Therefore, the EU and Arab countries/regions both agree on managing environmental noise, but from a legislative perspective, their frameworks are remarkably different. The EU’s comprehensive, detailed, enforceable regulations that are public health-based, noise mapping, and monitoring can serve as a practice for Arab countries/regions. In this comparison, we recognize a critical void in the Arab world where environmental noise pollution legislation is limited in structure and detail compared with that of the EU. As with Morocco, successfully managing environmental noise pollution by adopting recommendations along with best global practices to establish specific sound limits, health considerations, and valid noise mapping systems is essential.

### 4.3. Enforcement Mechanism and Penalty Framework

The legal framework imposes very high fines for noise-control violations. Specifically, 1000 to 20,000 dirhams for first offenses and 1000 to 40,000 dirhams plus imprisonment of between one day and thirty days for repeat offenses [43]. Given those fines, one would understand that the Moroccan legislation takes noise violations very seriously. After all, enforcement remains grossly deficient. Legally, for example, the law sets certain time restrictions: no noise-making options shall be allowed after 9 PM and no noise-making construction activity on a Sunday. Concerning enforcement, from the comparative study of counterparts and through best practices, some major concerns arise hindering the realization of legal statutes. Morocco is not exactly in a position to have the strictest noise limits that allow clearly laid provision measures, for enforcement purposes, to be laid down with respect to noise sources.

Morocco’s administrative structure supports the complexity of 12 regions and the difficulty in coordinating noise policy implementation, which extends beyond limited financial resources into the realm of complex multilevel governance. The increased complexity of institutions in Morocco is exaggerated by the lack of specific economic instruments to address noise pollution in Moroccan legislation, which have begun to emerge in countries/regions with more evolved noise control policies.

Morocco’s noise control involvement in the overall environmental management framework indicates both constraints and prospects. For example, Morocco’s economic instruments for the protection of the environment are varied, including water abstraction fees, electricity pricing, and motor vehicle tax systems. However, few economic instruments exist to control noise pollution. Environmental control policies and directives are limited by a lack of economic incentives to stimulate noise reduction and economic disincentives against excessive generation of noise. The legal framework is hampered by a lack of public awareness and opportunities for public involvement. Unlike the more developed environmental pollution control systems, which have public participation strategies and policies for environmental consultation, noise control in Morocco is restricted by limited opportunities for public involvement in noise control policy, implementation, and enforcement. This is noteworthy because noise is inherently a community problem that requires communities to provide knowledge and ongoing involvement to facilitate noise management.

## 5. Strategic Recommendations for Noise Control Policies

Noise pollution in Morocco calls for a comprehensive and precautionary noise control policy that recognizes both immediate needs and long-term strategic objectives to address the effects of noise on the community. The following recommendations constitute a multifaceted scheme for addressing the management of noise control, and draw from regional experiences as well as relevant international experiences and best practices.

- **Setting noise limits for different zones:** The urban structure of Morocco is fundamentally different from that of European cities—residential areas border commercial zones, traditional markets open late, and tourism areas are mixed with local community areas. The new noise limits must be differentiated to recognize these truths: the limits should be higher at night (10 PM–7 AM in entirely residential areas), graded in mixed-use tourist areas like Marrakech medina, where traditionally oriented storytelling, music, and market areas are intangible heritage, and selective measures may be made about spaces of cultural interest. Industrialized zones around the residential areas, also have to be provided with mandatory buffer zones and retrofitting provisions. Diesel generator and agricultural machinery activities are particularly problematic in rural and peri-urban regions and need purpose-specific rules and regulations, but not the same regulations as in the city. The initial cost of setting up noise-monitoring and control systems will be very high. However, in the long run, they will be very good for the health of the public and their quality of life, thus making them worth the price.
- **Noise mapping and monitoring systems:** Marrakech, Fez, Casablanca, and Rabat should be the first cities to embark on noise mapping. Mapping is expected to determine clashes between tourism infrastructure (clubs active up to 5 AM) and residential neighborhoods, gauge the level of noise generated by generators in electricity-deficient zones, and gauge the amount of noise generated by construction activities along the expanding highway system in Morocco between the Atlantic and Mediterranean routes. In contrast to generic mapping programs, the Moroccan approach needs to incorporate the population of mobile tourism and seasonal changes in the noise exposure.
- **Public awareness of noise pollution:** public awareness campaigns should focus on the vulnerabilities reported in schools, hospitals, and low-income districts near the transport lines. Due to the multilingual situation in Morocco and the countryside/urban disparity, the campaigns should be conducted using Arabic, the Amazigh language, and French in mosques, schools, local NGOs, and not only in the urban media.

▪ **Strengthening legislative enforcement:** Morocco’s 12-region administrative structure and municipal governance model require decentralized enforcement with central oversight. It is not enough to improve the fines provided under Law No. 11-03, which still fall between 1000 and 40,000 dirhams without the appropriate personnel training and adequate equipment. Based on this, the recommendations postulate the establishment of the regional noise enforcement bodies in the context of the current environmental agencies, the introduction of citizen reporting systems similar to the French one through WhatsApp and mobile applications adapted to the high levels of mobile usage in Morocco, and prioritization of enforcement based on empirically documented hotspots. Thus, Morocco would need to invest in the training of enforcement officers, public awareness, and technology-based monitoring to strengthen the law. Managing costs and tracking enforcement in high-risk urban areas can be accomplished via a phased approach to enforcement.
▪ **International cooperation and technical assistance:** The fact that Morocco does not have a systematic system of noise measurement highlights the need to build capacity, which goes beyond the acquisition of equipment. Strategic collaboration with European countries/regions (Germany, France, Sweden) must not only focus on training domestic technicians, customization of noise-mapping software to the urban structure of the country, and finding low-cost monitoring-based solutions that would fit in municipalities with limited resources. The challenges faced by Morocco could be effectively met by implementing real-time monitoring systems, such as those installed in cities in Europe, to measure the noise of generators in areas where power is not as reliable, and to police building sites during the rapid growth of large cities.

In recognition of the nascent state of noise pollution management in Morocco, the following phased roadmap is essential:

**Phase 1 (1–3 years)**: The first stage should involve a strict baseline test (which will involve carrying out pilot noise-mapping in big cities). At the same time, it should support specific awareness, conduct the audit of the gaps in the implementation of Law No. 11-03, and develop a course on training a preliminary group of enforcement authorities. In this undertaking, the formulation of Morocco-specific noise-testing protocols that specifically take into account the sources of generators, construction activity, and tourist sources is required.

**Phase 2 (4–7 years)**: The second stage will be performed when the noise-mapping is extended to all urban centers that have a population of more than one million people. Enforcement units will be created in the regions, and zone-specific regulation limits will be implemented to indicate the heterogeneous land-use structure peculiar to Morocco. It should introduce citizen-reporting systems, make surveillance democratic, and implement noise-controlling standards in the system of licensing of tourism enterprises and approval of the development projects in cities. Lastly, the program shall establish international technology-transfer relationships to acquire the best solutions.

**Phase 3 (8–10 years):** The third step will lead to coverage over the entire country, the reconciliation of WHO guidelines, and cultural specificity. This step will place Morocco at the forefront of the discourse on noise management in the Arab world, which is at present in Category 3 (Basic Framework). The experience gained during this time will be exploited to transfer the knowledge to adjacent states, and the noise control systems will be considered in the overall environmental and public-health systems.

This customized approach that considers the duality of Morocco as both a fast-modernizing economy and one of the leading tourism destination countries/regions. A proper noise-controlling system needs to be able to protect the health of people, preserve cultural heritage, and support sustainable economic growth. This makes the recommendations go beyond generic best practice and face some acute problems on the ground, including nocturnal nuisance created by wedding halls, sleep deprivation created by tourist infrastructure, noise in under-serviced districts created by generators, and the exigency of introducing a nationwide noise-mapping system into a country where cities have already surpassed the internationally recognized safety limits.

This systematic review recognizes various significant limitations that put its findings and scope into context. The first limitation is the low number of peer-reviewed studies from Morocco, which could impose some limitations on obtaining primary data from the literature. Thus, it limits the depth of empirical evidence available. The second issue is that some references contain non-peer-reviewed conference papers and grey literature because of the lack of primary sources, yet all of them have been verified through rigorous methodologies. The third limitation is that Morocco does not have a national noise mapping system, which makes it difficult to conduct spatial analysis and compare cities with one another. The limitations were dealt with by carrying out comprehensive searches for grey literature from UNECE, WHO, and RGPH sources in conjunction with PRISMA-compliant dual-reviewer validation. It is recommended that future research focus on nationwide noise mapping, longitudinal health impact studies, and standardized monitoring protocols as ways of bridging these gaps.

## 6. Conclusions

In conclusion, noise pollution in Morocco has become an increasingly severe environmental challenge that requires urgent attention from both policymakers and the public. Increased noise levels are a result of rapid development, beginning with Morocco’s major cities but extending into the countryside and smaller towns. Increased noise impacts society at every level, and the costs, although difficult to quantify, are considerable in terms of human health and impacts on the environment. In this systematic literature review, we show that the current legalistic framework addressing noise pollution is an encouraging first step, yet the situation now calls for much more, since legal lacunas are there, insufficient noise levels are prescribed, they are not enforced where they are, and even with legislation, there is no compliance or operational support on which to pursue.

The comparison of noise management policies internationally, with more emphasis on the EU’s policies, points to an enhancement in the Moroccan response to noise problems through an elaborate regulatory framework that uniquely identifies various roles, comprehensive legislation, legal requirements linked to comprehensive noise mapping, and safeguards against unintended consequences, including tight administrative monitoring of the regulation. A complete noise-related policy must be put forward by Morocco if it is going to protect the health of the population and lead to better urban living experiences.

The eleven recommendations of the systematic literature review should include the identification of a source of noise, decibel standards for noise limits, noise mapping, enforcement, and serious public engagement and awareness to close the gaps, achieving stillness for some within a noisy environment. With a serious approach to the management of noise pollution in Morocco, the country can establish its national and global visibility as a leader in helping to promote national public health, protect against the dangers of noise, and create healthier and more sustainable urban environments.

## Figures and Tables

**Table 1:** The status of noise pollution legislation in Arab League countries/regions.

Country	Environmental Law or Decree	Specific Noise Legislation	Permissible Noise Limits	Measurement Protocols	Technical Guidelines	Noise Definition	Additional Notes	Reference
Algeria	✅ Yes	✅ Yes	✅ Yes	✅ Yes	❌ No	✅ Yes	Developed legislation in 1980s; 2-zone classification	[50,52]
Oman	✅ Yes	✅ Yes	✅ Yes	✅ Yes	✅ Yes	✅ Yes	Advanced technical guidelines and protocols	[54]
Saudi Arabia	✅ Yes	✅ Yes	✅ Yes	✅ Yes	✅ Yes	❌ No	Most advanced technical framework in the region	[60,63]
Qatar	✅ Yes	✅ Yes	✅ Yes	❌ No	✅ Yes	✅ Yes	Comprehensive noise level estimation guidelines	[68,75]
United Arab Emirates	✅ Yes	✅ Yes	✅ Yes	✅ Yes	❌ No	✅ Yes	Federal-level noise regulations	[58]
Jordan	✅ Yes	✅ Yes	✅ Yes	✅ Yes	❌ No	✅ Yes	Comprehensive noise policy framework	[70,76]
Egypt	✅ Yes	✅ Yes	✅ Yes	✅ Yes	❌ No	❌ No	Established noise limits through specific legislation	[53]
Iraq	✅ Yes	✅ Yes	✅ Yes	❌ No	❌ No	❌ No	Pioneer in noise legislation (mid-1960s); 11-zone classification	[49,71,73]
Morocco	✅ Yes	✅ Yes	❌ No rigorous limits	❌ Limited	❌ No	✅ Yes	Law 11-03 (2003); penalties 1000–40,000 dirhams	[43,44,45,46,47,48]
Syria	✅ Yes	❌ No	✅ Yes	❌ No	❌ No	❌ No	Limits established through environmental law	[61,77]
Tunisia	✅ Yes	✅ Yes	✅ Yes	❌ No	❌ No	❌ No	Developed legislation in the 1980s; limits via environmental law	[51,67,78,79,80]
Djibouti	✅ Yes	❌ No	❌ No	❌ No	❌ No	✅ Yes	Environmental law only	[72]
Somalia	✅ Yes	✅ Yes Partial	❌ No	❌ No	❌ No	✅ Yes	Both environmental and noise legislation	[81,82]
Bahrain	✅ Yes	❌ No	❌ No	❌ No	❌ No	❌ No	Environmental law only, no noise provisions	[57]
Comoros	✅ Yes	❌ No	❌ No	❌ No	❌ No	❌ No	Noise not mentioned in legislation	[83,84,85]
Kuwait	✅ Yes	❌ No	❌ No	❌ No	❌ No	❌ No	Environmental law only	[55,64]
Lebanon	✅ Yes	❌ No	❌ No	❌ No	❌ No	❌ No	Environmental law only	[66]
Libya	✅ Yes	❌ No	❌ No	❌ No	❌ No	❌ No	Noise not mentioned in legislation	[69]
Mauritania	✅ Yes	❌ No	❌ No	❌ No	❌ No	❌ No	Noise not mentioned in legislation	[86]
Palestine	✅ Yes	❌ No	❌ No	❌ No	❌ No	❌ No	Environmental law only	[59]
Sudan	✅ Yes	❌ No	❌ No	❌ No	❌ No	❌ No	Environmental law only	[87]
Yemen	✅ Yes	❌ No	❌ No	❌ No	❌ No	❌ No	Environmental law only	[62]

**Table 2:** Overall performance of Arab League countries/regions in terms of noise pollution (22 Countries/regions).

	Countries	Percentage
Countries/regions with Environmental Laws	22/22	100%
Countries/regions with Specific Noise Legislation	11/22	50%
Countries/regions with Permissible Noise Limits	10/22	45%
Countries/regions with Measurement Protocols	6/22	27.3%
Countries/regions with Technical Guidelines	3/22	13.63%

**Table 3:** Arab leading countries/regions classified by their noise policy framework.

Framework Level	Country	Specific Features
**Category 1: Advanced Framework**	Saudi Arabia	▪ The most detailed system, including technical guidelines, measurement protocols, and day/night differences.
	Qatar	▪ Advanced noise level estimation guidelines and limits.
	Oman	▪ Technical guidelines and measurement protocols.
**Category 2: Intermediate Framework**	Algeria	▪ Specific legislation with limits and day/night periods.
	Jordan	▪ Comprehensive policy with measurement protocols.
	UAE	▪ Federal regulations with measurement protocols.
**Category 3: Basic Framework**	Egypt	▪ Specific legislation with basic limits.
	Iraq	▪ Pioneer status but limited technical development.
	Morocco	▪ Specific legislation, but it lacks rigorous limits.
**Category 4: Environmental Law Only**	Syria, Tunisia, and Somalia	▪ Limits through environmental law.
	12 other countries/regions	▪ Environmental law without noise provisions.

**Table 4:** Comparison of Noise Regulation of Morocco and the European Union.

Aspect	Morocco	European Union
**Specific noise legislation**	Yes [43]	Yes (Directive 2002/49/EC) [98]
**Permissible noise limits**	No rigorous limits [43]	Comprehensive limits [89,90,91,92,93,94,95]
**Noise mapping**	Absent [43]	Mandatory [89,90,91,92,93,94,95]
**Measurement protocols**	Limited [43]	Standardized (ISO-based) [89,90,91,92,93,94,95]
**Action plans**	Non-existent [43]	Required [89,90,91,92,93,94,95]
**Health impact assessment**	Minimal [43]	Comprehensive [89,90,91,92,93,94,95]

**Table 5:** Environmental noise regulation frameworks of Arab countries/regions compared with those of the European Union.

Category	Arab Countries/Regions (Including Morocco)	European Union
**Legislative Framework Structure**	Fragmented laws	Comprehensive
**Specific Noise Legislation**	Limited (a few countries/regions)	Detailed, mandatory
**Permissible Noise Limits**	Less specific	Precise, defined
**Zone-Specific Regulations**	Limited zones	Clear zone-based
**Health Impact**	Nuisance focus	Public health integrated
**Noise Mapping Systems**	Largely absent	Comprehensive mapping
**Monitoring/Assessment**	Limited monitoring	Mandatory regular
**Guidance Docs**	Limited scope	Comprehensive
**Enforcement**	Weak enforcement	Strong, detailed

## Data Availability

The datasets used and/or analyzed during the current systematic review are available from M. El Malki on reasonable request.

## Supplementary Materials

The following supporting information can be downloaded at: https://www.mdpi.com/article/10.3390/ijerph23010073/s1, File S1: Systematic Review of Noise Pollution in Morocco: Regulatory Frameworks, Urban Impacts, and Policy Recommendations. Reference [99] is cited in the Supplementary Materials.
