# Supplementary material for: Systematic Review of Noise Pollution in Morocco: Regulatory Frameworks, Urban Impacts, and Policy Recommendations
Source: Int J Environ Res Public Health. 2026 Jan 4;23(1):73. doi: 10.3390/ijerph23010073 (PMC12840975; doi:10.3390/ijerph23010073)
Supplement: Supplementary file 1 [file ijerph-23-00073-s001.zip › ijerph-3930201-supplementary.pdf]

Supplementary Material: Systematic Review of Noise Pollution in Morocco: Regulatory Frameworks, Urban Impacts, and Policy Recommendations

Mohamed El Malki [1,*], Ali Khettabi [1], Felipe A. de Figueiredo [2], and Mohammed Serrar [3]

[1] Laboratory of Materials, Waves, Energy and Environment, Department of Physics, Faculty of Sciences, Mohammed First University, Oujda 60000, Morocco; m.elmalki@ump.ac.ma (M. El Malki); a.khettabi@ump.ac.ma (A. Khettabi)
[2] National Institute of Telecommunications (Inatel), Santa Rita do Sapucaí, MG 37536-001, Brazil; felipe.figueiredo@inatel.br
[3] Faculty of Legal, Economic, and Social Sciences, Mohammed First University, Oujda 60000, Morocco; mohammed.serrar@ump.ac.ma
* Correspondence: m.elmalki@ump.ac.ma

This supplementary material aims to enhance transparency and completeness in reporting as per PRISMA guidelines, supporting reproducibility and scientific integrity.

Table S1. The PRISMA checklist status of noise pollution legislation in Arab League countries.

Section and Topic	Item #	Checklist item	Location where item is reported
**TITLE**			
Title	1	The manuscript title directly identifies the report as a systematic review.	Title page
**ABSTRACT**			
Abstract	2	The abstract outlines the background, objectives, methods, major findings, and policy implications, based on PRISMA abstract checklist requirements.	Page 1
**INTRODUCTION**			
Rationale	3	The introduction reveals some ongoing gaps in the academic and legislative efforts in Morocco, with respect to noise pollution, public health, and the regulatory context, and explains the need to conduct the review.	Page 1 to 4
Objectives	4	The current research aims to explore the issue of increasing noise pollution in Morocco, in relation to its impact on the major cities, legal frameworks, health, and barriers towards implementation.	Page 4
**METHODS**			
Eligibility criteria	5	The inclusion criteria included: (i) studies covering the topic of noise pollution in Morocco, including the underlying impact of the noise in urban areas, laws, regulations, or health impacts of noise pollution. (ii) articles and institutional reports published between 2003 and 2025. (iii) quantitative studies and qualitative studies that were relevant to policy, adverse effects of the noise	Page 5 to 7

Section and Topic	Item #	Checklist item	Location where item is reported
		in cities, or its effects on regulations, or their health issues.<br>Exclusion criteria included (i) studies not relevant to noise pollution or those focusing solely on other forms of environmental pollution. (ii) Non-English or non-French studies, except when containing important or unique data, and (iii) reports lacking empirical data or not addressing policy, regulatory, or urban noise components.<br>Studies were grouped into two main arms for synthesis: (i) Moroccan studies (n=23) focused on national legislation, urban noise levels, and health impacts. (ii) Moroccan legal documents (n=6), international, comparative international sources, divided into Arab regional studies (n=48), EU legislative frameworks (n=13), standards (n=6), and methodological references (n=2).<br>Data were synthesized thematically: urban impacts, regulatory deficiencies, health effects, and comparative analysis with international best practices.	
Information sources	6	The main search sources, which were used, included Scopus, Web of Science, and Google Scholar. The institutional and organizational reports were of the United Nations Economic Commission, the World Health Organization (WHO), the Moroccan national agencies, which include the High Commission of Planning (HCP), and national censuses, plus the local environmental agencies and legal document repositories.<br>Other resources included the regional comparative studies of the Arab League states (systematic searches of government and legal databases), reference lists of the used articles, and official regulatory documents were included in the comparative analysis of the European legislative material. The last systematic search and consultation of sources was completed in August 15, 2025. Specifically: identification/screening (15 January – April 2025), eligibility (full-text, May – June 2025), included studies (extraction/synthesis, July–15 August 2025), using the full Boolean strings and 2003–2025 inclusion criteria.	Page 6
Search strategy	7	The systematic search plan used a combination of various databases containing authoritative reports, linguistic filtering, and specific keywords that routinely and selectively capture all relevant literature regarding noise pollution in Morocco.<br>The searching formula, which was used in all databases, was the following:<br>• Scopus (Elsevier): TITLE-ABS-KEY (("noise pollution" OR "urban noise") AND ("Morocco" OR "Moroccan cities") AND ("public health" OR "health impact" OR "noise regulations"))<br>• Web of Science Core Collection: (("noise pollution" OR "urban noise") AND ("Morocco" OR "Moroccan cities") AND ("public health" OR "health impact" OR "noise regulations"))<br>• Google Scholar: ("noise pollution" OR "urban noise") AND ("Morocco" OR "Moroccan cities") AND ("public health" OR "health impact" OR "noise regulations")<br>The search strategy to track relevant papers included:<br>• Developed comprehensive keyword sets related to noise pollution in Morocco, including terms such as "noise pollution Morocco," "urban noise," "noise regulation," and "health impacts."<br>• Combined keywords using Boolean operators (AND, OR) tailored for each database.<br>• Applied filters apply or exclude results to articles published from 2003 to 2025 (except in special cases, such as legislation reports), and are limited to peer-reviewed articles and institutional reports in English, French, and Arabic in some special cases related to regulation documents.<br>• Manual search of the organizational websites (i.e., UNECE, WHO, the Moroccan government, etc.) of grey literature and policy documents. - Inspected reference lists of obtained studies to determine other relevant literature.	Page 6 to 7

Section and Topic	Item #	Checklist item	Location where item is reported
		• Comprehensive records were maintained detailing all search terms, databases, search times, and filters to ensure transparency.<br>• Titles and abstracts were initially screened, and then full texts were evaluated by two independent reviewers using predefined inclusion criteria focused on the effects of noise pollution in Morocco, related health outcomes, and the regulatory framework.	
Selection process	8	Inclusion Criteria: The studies had to be either a peer-reviewed article or an institutional finding that dealt with noise pollution in Morocco and specifically on the control framework systems, the level of noise pollution in the city, health consequences, or the policy action response. Qualified studies included empirical quantitative or qualitative designs that were published in 2003 onwards. Clutch studies referring to comparative analyses of the Arab regional or international structure were also taken into account.<br>Exclusion Criteria: The studies that were not directly involved with noise pollution or perceived different environmental pollutants, non-empirical reports, and those published in languages other than English and French without critical data were excluded. Articles that did not provide enough information regarding methodology or policy-relevant or city-related impacts were also filtered.<br>Screening Process: The databases of Scopus, Web of Science, and Google Scholar were used to identify a total of 23 Moroccan studies, and other comparative studies were used as complementary to the specific institutional type documents of UNECE, WHO, and Moroccan governmental agencies. Initial title and abstract screening were done by two reviewers independently so that they could eliminate irrelevant records. Reviewers independently applied the predefined criteria to the full texts of potentially eligible studies. Any disagreements or uncertainties between the two reviewers were resolved through discussion, and if necessary, a third reviewer adjudicated.<br>Automation Tools: No automation was applied during screening or selection, and everything was completed manually to ensure thorough coverage and control of quality.	Page 6 to 7
Data collection process	9	Data extraction was conducted independently by two reviewers who systematically collected relevant information from each included report using a standardized form. To ensure accuracy and consistency, a third reviewer cross-validated the extracted data to resolve any discrepancies. Extracted data included study objectives, measurement techniques, urban noise levels, and assessments of health and legal perspectives. Any computations applied in data collection were carried out manually, adding to the methodological rigor and dependability.	Page 7
Data items	10a	All the included outcomes associated with noise pollution within the realm of Morocco, their health effects (cardiovascular disease, sleep disturbance, stress, cognitive impairment), regulatory policies, urban noise pollution, and policy recommendations. Users gathered publications published between 2003 and 2025 and included the different measures of noise levels (L90, Leq), daytime and nighttime measurements, and qualitative and quantitative comparisons. Although the review aimed to include all relevant findings across different outcomes, limitations arose due to inconsistent study quality, limited clinical measurement information, and a lack of longitudinal health data. We used a standardized process of data extraction and conducted it by personnel reviewing transactions, such that outcomes of various study designs are comprehensively yet realistically captured.	Page 6 to 7
	10b	Data on population vulnerability, legislative and intervention context, the method of measurement, and research funding have been made available in a systematic way to enhance the outcome data, but noted limitations on the completeness and quality of the data contained in the comprising studies have been discussed.	Page 6 to 7
Study risk of bias	11	To ensure objectivity and reliability, each study's risk of bias was assessed independently by two reviewers. Disagreements were	Page 7

Section and Topic	Item #	Checklist item	Location where item is reported
assessment		resolved through discussion or, if needed, by consulting a third reviewer. To measure the quality of the studies, a standardised assessment tool or checklist was used, considering the study design, completeness of the data, reliability of the measurements, and its attribution to noise-pollution effects.<br><br>No automation tools were reported as being used in the risk of bias assessment process. The procedure was performed manually, allowing experienced evaluation to address the possible biases due to the variability in the quality of the studies, insufficiency of the clinical data, and the limitations inherent to measuring the environment and health outcomes.	
Effect measures	12	For each outcome in the review, effect measures used in the synthesis and presentation of results included:<br>• Continuous noise-exposure measurements in decibels (dBA) with quantitative and qualitative metrics: L90, Leq as well as day-night differences;<br>• Comparisons with international non-stated noise limits: prevalence rates or proportions of noise-associated health effects: cardiovascular disease, sleep disturbance, stress, and cognitive impairments;<br>• Descriptive statistics and qualitative summaries of regulatory frameworks, legislative gaps, and policy recommendations;<br>• The spatial and temporal differences are shown as noise maps or monitoring-data visualisations where possible.<br>The review did not report the use of standardized effect size measures like risk ratios or mean differences due to heterogeneous study designs and outcome types. Instead, the review included quantitative data on noise measurements with qualitative data on policy assessment and health-impact evaluation in order to create a complete synthesis of evidence.	Page 7 to 11
Synthesis methods	13a	Synthetic eligible studies were appropriately stratified into two main arms: Moroccan studies (n=23), including legislation, urban noise, and health studies; and multiple Arab-regional studies (n=48), EU legislative frameworks (n=13), standards (n=6), and methodological references (n=2).<br>The decisions were made by tabulating the characteristics of studies and types of interventions, and estimating outcomes and contrasting these with the pre-identified sets of synthesis, namely, urban impacts, regulatory inadequacy, health outcomes, and comparison of advancements with international reference points.<br>This was an arranged grouping, which enabled a logical thematic synthesis in harmony with the purpose of the review.	Page 7
	13b	Preparation of data to be presented and synthesized entailed the extraction of all relevant quantitative and qualitative data related to noise pollution from the studies conducted so far that met the eligibility criteria. Missing summary statistics were addressed by including reported noise-level measures (e.g. L90, Leq, day-night variations) were treated as continuous data where it was feasible. Where there were gaps or discrepancies in the summary measures and where the decisions became incomplete, synthesis of descriptive statistics and qualitative context data was carried out narratively. No complex data conversions or imputation techniques were applied due to variability in study designs and data reporting formats. Standardized data extraction forms were used by independent reviewers to systematically capture study aims, noise measurement methods, outcomes, and policy implications, ensuring consistent data collection.	Page 7 to 20
	13c	Individual study and synthesis findings were matched up into tabulation forms, based on data-extraction sheets, the studies according to the geographic location, outcome domains (urban noise levels, regulatory frameworks, health impacts), and measurement modalities. Visual presentations included a summary of the narrative and tables that compared the noise-exposure measurements with international measures. The review incorporated thematic grouping of data (urban impacts, regulatory deficiencies, health effects, comparative analyses) rather than quantitative graphs or meta-analytic visuals due to heterogeneous data and the absence of uniform effect size measures.	Page 7

Section and Topic	Item #	Checklist item	Location where item is reported
	13d	The thematic synthesis approach adopted by the authors fits the heterogeneity of the body of empirical literature addressing noise pollution in Morocco well. Due to the variety of design typologies used in the main studies and the overall inability of the literature to provide any standardized measure of effect sizes and any consistent measure of outcome measures, a classical meta-analytic aggregation was considered unsuitable. This is why the authors comparatively synthesized quantitative noise measurements descriptively, such that these data sets are interconnected with qualitative assessments of current regulatory mechanisms and health implications. This integrative narrative synthesis facilitated a comprehensive understanding of policy gaps, environmental impacts, and health effects of the locals that were specific to the urban setting in Morocco. The rationale behind the decision to apply thematic synthesis was that it was necessary to quantitatively describe all manifestations of evidence and condense actionable policy advice, and not statistical condensation given by nature.	Page 6 to 21
	13e	The review did not perform formal quantitative assessments of heterogeneity (in terms of subgroup tests, meta-regressions, etc.) due to the heterogeneity of study designs, outcomes, and measures. Instead, heterogeneity was explored qualitatively by grouping studies by salient features, such as by geographic location (primarily the containing larger Moroccan cities), area of outcome (levels of urban noise, legislative frameworks, health), and intervention or policy context. Thematic synthesis was carried out with full consideration of the differences in measuring methods, the vulnerability of the population portrayed by measurements, and the legislative context. Thematic synthesis considered variability in noise measurement methods, population vulnerability, and legislative contexts as potential sources of heterogeneity influencing findings.	Page 8 to 22
	13f	No formal sensitivity analyses were conducted, which is also in line with the narrative and thematic method of synthesis. However, the rigor of the study was complemented by adopting rather strict inclusion criteria that constrained the dataset to national research that was closely related to the context of noise pollution in Morocco and to its policy context. This methodology has allowed the analysis to be relevant and have methodological integrity.	-
Reporting bias assessment	14	The authors clearly point out the limitations related to reporting biases, due to the scarcity and the agedness of the data, to the inconsistency of research, and to the possible non-publication of the research that produces no effect or unfavorable outcomes in the context of Morocco.<br>This systematic review recognizes various significant limitations that put its findings and scope into context. The first limitation is the low number of peer-reviewed studies from Morocco, which could impose some limitations on obtaining primary data from the literature. Thus, it limits the depth of empirical evidence available. The second issue is that some references contain non-peer-reviewed conference papers and grey literature because of the lack of primary sources, yet all of them have been verified through rigorous methodologies. The third limitation is that Morocco does not have a national noise mapping system, which makes it difficult to conduct spatial analysis and compare cities with one another. The limitations were dealt with by carrying out comprehensive searches for grey literature from UNECE, WHO, and RGPH sources in conjunction with PRISMA-compliant dual-reviewer validation. It is recommended that future research focus on nationwide noise mapping, longitudinal health impact studies, and standardized monitoring protocols as ways of bridging these gaps.<br>Although no formal statistical methods (such as funnel plots) to identify publication bias were used, the review team removed bias by conducting exhaustive searches of databases, including institutional reports in addition to peer-reviewed literature, and cross-checking information sources. These steps aimed to capture a broad evidence base and identify grey literature that might contain unpublished or underreported results.	Page 7
Certainty assessment	15	Certainty in evidence was assessed qualitatively by evaluating study design robustness, consistency of findings, and relevance to the Moroccan context. The authors acknowledge the limitations that are caused by a small sample, a lack of longitudinal data,	Page 7 to 9

Section and Topic	Item #	Checklist item	Location where item is reported
		and different methodological directions. The narrative synthesis identifies areas of strength (e.g., data indicating noise levels in cities go above internationally agreed-upon limits) and those areas requiring additional empirical research (e.g., much more detailed clinical trials on the effects of health).	
**RESULTS**			
Study selection	16a	The search and selection procedure involved a thorough search of research and legislative texts concerning noise pollution in Morocco and similar contexts. A total of 98 records were discovered, and these included: Moroccan research, local Arab referencing, EU laws, and legislation. An additional screening and eligibility check were used to narrow the corpus to the most relevant entries, and 23 studies in Morocco were obtained, plus a selection of comparative sources of legislation to undergo lengthy examination. Each of these procedural stages is outlined in the flow diagram.	Page 5 to 7
	16b	The studies that may seem to fit the inclusion criteria, but that were not included El Ghazi et al. [1] that focus more on air pollution, particularly nitrogen dioxide generated by traffic sources, instead of focusing on noise pollution. El Husseiny et al [2] have come so far as to represent a conceptual and analytical literature review and not the conduction of empirical noise pollution data specific to the Moroccan environment or a comprehensive approach to environmental noise. Belyagou et al. [3] pay attention to the aspects of the territorial processes and formation without presenting any empirical evidence of noise pollution or its effects. In addition, no exploration or information on noise pollution was observed in the study of Lamsiah and Bentalha that focused on tourism and diplomacy [4]. In [5], noise is mentioned in the context of the mitigation measures, without the empirical research or noise-pollution-oriented research being conducted, and, thus, does not meet the inclusion criteria of empirical studies of noise.<br><br>References:<br>[1] El Ghazi, I.; Berni, I.; Menouni, A.; Amane, M.; Kestemont, M. P.; El Jaafari, S., Exposure to air pollution from road traffic and incidence of respiratory diseases in the city of Meknes, Morocco. Pollutants 2022, 2, (3), 306-327. https://doi.org/10.3390/pollutants2030020<br>[2] El-Husseiny, M.; Mashaly, I.; Azouz, N.; Sakr, N.; Seddik, K.; Atallah, S., Exploring sustainable urban mobility in Africa-and-MENA universities towards intersectional future research. Transportation Research Interdisciplinary Perspectives 2024, 26, 101167. https://doi.org/10.1016/j.trip.2024.101167<br>[3] Belyagou, Y.; El Hajri, A.; Mellouki, A.; El Abdioui, S., Déterminants de la dynamique territoriale: Cas de la région Fès-Meknès. Alternatives Managériales Économiques 2022, 4, (2), 253-273 https://doi.org/10.48374/IMIST.PRSM/ame-v4i2.32194<br>[4] Lamsiah, A.; Bentalha, B., Dakhla: A Growing Touristic Destination with a Diplomatic Sway. Alternatives Managériales Économiques 2022, 4, 94-115. https://doi.org/10.48374/IMIST.PRSM/ame-v1i0.36936<br>[5] African Development Bank Group, Project to Strengthen Infrastructure Between Casablanca and Marrakesh - Environmental and Social Impact Assessment Summary 2015, 1, (1), 1-50. Availible from https://www.afdb.org/fileadmin/uploads/afdb/Documents/Environmental-and-Social-Assessments/Maroc-Projet_de_renforcement_des_infrastructures_entre_Casablanca_et_Marrakech-Résumé_EIES-_EN_-_08_2015.pdf	-
Study characteristics	17	The review included a total of 98 full-text records were analyzed and categorized by type and source. Peer-reviewed articles comprised 52%, conference papers 31%, reports 9%, and legal or regulatory texts 8%, showing that academic publications dominate but grey literature still plays a major role. Journal articles were mostly retrieved from Scopus and Web of Science, while governmental and legal documents were gathered from institutional and public online portals. Limited tracking of database-specific contributions was noted as a methodological constraint.<br><br>Publication trends demonstrated a significant rise in research output, with 67% of studies produced between 2016–2025, reflecting growing regional and national interest. Despite this, Morocco remains among the least productive countries in the Arab League, contributing only 1.9% of regional output, indicating a persistent expertise gap that affects evidence-based	Page 7 to 9

Section and Topic	Item #	Checklist item	Location where item is reported
		policymaking, although recent acceleration is promising.<br>Regarding language, 79% of documents were published in English, 17% in French, and 4% in Arabic, reflecting the dominance of English in scientific publications and the use of French/Arabic for regulatory and institutional materials. All references were accessible online.<br>Institutionally, most Moroccan empirical studies originate from a small cluster of university laboratories in physics, engineering, environmental science, and public health. This concentration highlights progress but also the need to broaden national institutional participation in noise research and policy support.	
Risk of bias in studies	18	The paper acknowledges variability in the quality of the included studies. Several studies lacked detailed information on noise-level quantification or had no strong health-outcome data; likewise, noise-pollution datasets were in some cases sparse or out of date, none of which were reported as limitations to the study. Concerted efforts were made to ensure no irrelevant studies were incorporated or sources were cross-checked to be certain of avoiding bias in the study. There was, however, limited funding and availability of primary data, which meant that the depth of the analysis and the extent were subjected to significant limitations.<br>The review also highlights the difficulties of measuring the direct clinical health implications by referring to the lack of objective physiological measurement methodology in the reviewed literature. As such, even though the review provides the risk of bias related to methodological variability, incomplete data, and the insufficient rigor of measurements, it does not go further toward the application of a formal risk-of-bias scoring (e.g., RoB 2 or ROBINS-I). Rather, an evaluation by narrative that was complemented by a dual-reviewer process helps mitigate bias.	-
Results of individual studies	19	In methodological terms, there was a strong variety of studies, as some were quantified at noise levels, increasing the influence of the intervention, others at the health levels, and in general, reducing the chances of establishing uniform summary statistics within the corpus. In fact, most reports did not present quantitative estimates of the effects in granular form or confidence intervals; e.g., in most cases, it was through the narratives or generalized associations based on WHO evidence, not quantitative estimates of the effects using a study-specific statistic. All of the noise-measurement literature contained mainly descriptive measurements of exposure (e.g., dBA levels, Lden values) and no considerations of inferential statistics or effect-size estimates related to their health outcomes.<br>Taking into consideration the strong heterogeneity, as well as limited access to similar quantitative information, the authors did not intend to perform a meta-analysis, as a result of which it was not possible to report combined estimates of effects of a simulation and indicators of accuracy. The synthesis did not, therefore, attempt to add structured tables of per-study statistics or forest plots to make the synthesis post hoc. Finally, the available data in the review was presented in a narrative or generalized way, avoiding detailed per-study quantitative tables or visualization to provide a notion of granularity in communicating the effects of a study at actual, not only larger, but communicative scales.	-
Results of syntheses	20a	• There is variability in study quality, with some lacking detailed noise measurements or comprehensive health outcome data.<br>• Research is also limited due to the inaccessibility of primary data, funding limitations, and the heterogeneity in methods, where only sparse information on noise pollution exists, mostly obsolete.<br>• Lack of unified noise measurement guidelines and tools to implement noise limits and control raises a question of possible bias when reporting and describing conclusions.<br>• The majority of studies focus on descriptive measures of exposure without using rigorous statistical estimates of the effect, as well as direct clinical health-impact measurements.<br>• The literature synthesis is mostly narrative and captures the heterogeneous nature and breadth of the existing studies instead	Page 2 to 6 & 7 to 10

Section and Topic	Item #	Checklist item	Location where item is reported
		of assigning quantitative risk-of-bias scores to each study.<br>• Regional and institutional barriers, such as ineffective legislation and a lack of enforcement capacity, indirectly affect issues related to data reliability<br>Collectively, these factors indicate that, although the studies analyzed provide valuable insights into the effects of noise pollution and Morocco's regulatory environment, methodological shortcomings, inconsistent data quality, and incomplete health effect indicators contribute to a quantifiable risk of bias. The review, thus, emphasizes the need for standardized procedures, supplementary data gathering, and effective regulatory efforts to strengthen research and policy creation moving forward.	
	20b	No statistical syntheses or meta-analyses were conducted for the reviewed noise pollution studies in Morocco. Consequently:<br>• No summary estimates, confidence intervals, or statistical heterogeneity measures for outcomes were reported.<br>• A meta-analysis was not feasible due to the heterogeneity of studies and insufficient quantitative data.<br>• Conclusions are expressed qualitatively through narrative synthesis, rather than through statistical pooling.<br>• Group and study result comparisons are described narratively, highlighting consistently higher noise levels in cities exceeding international safety thresholds, which are associated with health problems such as cardiovascular disease and sleep disorders<br>• Ruth tortuosity of effects that have been reported in single studies or rather the evidence base in general is invariably towards harmful health effects and regulatory deficiencies, which forms the outlook of noise pollution load.	-
	20c	No formal statistical inquiry was conducted in this work based on the known factors that could have caused heterogeneity between the study results of noise pollution in Morocco. The observed heterogeneity of the reviewed studies was qualitative due to the presence of the following factors:<br>• Diversity in Study Design and Methods: The studies included in the analysis used varying approaches, with the differences in approaches being noise-level measurements (applicable types include L90, Leq, etc.), and qualitative measures of the health effects and regulatory frameworks.<br>• This heterogeneity methodology makes results comparison and synthesis difficult. Variations in Geographic Focus: investigations were carried out in diverse cities, including Casablanca, Rabat, Fez, Marrakech, and Tangier in Morocco, and had different urban architecture, density, and sources of noise, causing regional differences in the research.<br>• Differences in Data Completeness and Quality: Noise data or full health-outcome data are not provided in some studies, which has led to variation in the reported effects.<br>• Uneven Regulatory and Environmental Frameworks: The variation in local applications, observation resources, and policies among cities over time poses a challenge to noise exposure levels and assessment results.<br>• Small Temporal and Longitudinal Data: Too often, the available data are cross-sectional or short-term, which lessens the ability to determine change over time or causal relationships.<br>• Variations in Sources of Noise and Urbanism: The differences in the vehicle traffic, construction, industrial noise, and nightlife vary by location and influence the study outcomes.<br>• Limitations caused by Sparse Financing and Research Capacity: Selected data collection and analysis rigor variability and gaps have been driven by insufficient in facility and research funding in Morocco.	Page 7 to 20
	20d	There were no sensitivity tests to determine the sensitivity of the results that were synthesized. Due to the characteristics of the review, which is mostly qualitative and narrative because of the heterogeneous study designs, low qualitative data, and lack of meta-analyses, statistical syntheses and sensitivity testing were not needed. The paper recognizes the constraints and heterogeneity in studies included in the paper, but failed to obtain formal sensitivity analyses, like eliminating selected studies, using different inclusion criteria, or examining the effect of methodological quality on the outcome. This is consistent with the	-

Section and Topic	Item #	Checklist item	Location where item is reported
		overall descriptive approach to synthesizing evidence on noise pollution in Morocco.	
Reporting biases	21	There was no formal evaluation presented on the risk of bias based on the non-reporting of results based to reporting biases by each synthesis being assessed. The review admits some limitations that pertain to data availability and research gaps, but the control of the reporting biases, namely, publication bias, selective outcome reporting, or missing data, is not addressed.<br>Key points include:<br>• The evidence base regarding noise pollution in Morocco is scattered and narrow, as some of the studies do not provide all data on the noise levels or health consequences.<br>• Comprehensive reporting and data completeness are limited by sparse, sometimes outdated data, insufficient funding, and constrained research resources.<br>• The review sought to minimize bias through comprehensive search strategies in multiple databases and by including regional, European, international, and Moroccan studies.<br>• No formal procedures like funnel plots, publication bias statistical tests, or a qualitative evaluation of selective reporting were reported.<br>• It takes into consideration risk due to underreporting of some outcomes or studies, but no systematic evaluation of bias based on missing results has been provided.	Page 6 to 16
Certainty of evidence	22	The research lacks formal, structured measurements on certainty or confidence in the body of evidence of each outcome as described by programs like Grading of Recommendations, Assessment, Development and Evaluation (GRADE). The synthesis of evidence is largely qualitative and narrative, as the studies on the subject are heterogeneous, and quantitative data are also very few and far between in the synthesis of statistics.<br>However, the paper explicitly addresses factors that determine confidence concerning what the evidence overall in Morocco says about the effects of noise pollution, including:<br>• Few studies explicitly quantify health effects by using strict methodologies, which minimizes some form of certainty.<br>• Despite discrepancies and missing data in noise indicators, health outcomes determination, and urban surveillance reduces the level of confidence.<br>• The dependency on the regional, international, and WHO recommendations complements local evidence but suggests indirect inference instead of direct recognition of causes.<br>• Regulatory and enforcement issues increase the failure of effective applications to noise control, hence the translation of evidence to policy.<br>• The review highlights the necessity to standardize the studies, have more longitudinal data, and validate exposure-response assessments to increase confidence in the evidence in the future.	Page 6 to 8
**DISCUSSION**			
Discussion	23a	The review substantiates that noise pollution in large cities in Morocco is often above the international norm of safety, thus leading to poor health conditions of cardiovascular disease, sleeplessness, and stress. This conclusion is in line with the World Health Organization's evidence that having chronic exposures of over 55 dB is harmful to community health.<br>The noise control regulatory environment in Morocco, which is mainly created under the implementation of Law No. 11-03, is characterised by an overall framework but lacks strict enforcement measures, specification of allowed noise levels, noise mapping, and uniform monitoring standards. This is unlike more superior frameworks, in other nations like Saudi Arabia, Qatar, the European Union (EU), and some Arab nations, where laws regarding noise pollution include technical guidelines,	Page 6 to 20

Section and Topic	Item #	Checklist item	Location where item is reported
		measurement guidelines, and specific day-night levels. Morocco ranks in the mid-range in terms of regional comparisons in terms of noise policy development, with significant disparities in technical implementation and enforcement of noise policy.<br>In contrast to certain other Arab nations that have stronger noise regulations, Morocco has a more flexible approach to noise as it is a part of environmental law and not treated independently as a public health issue.<br>The review has also not focused on the absence of major urban environmental inequalities, in which low-income neighbourhoods are disproportionate victims of noise harms and mitigating resources.<br>This trend is illustrated in other less developed nations, and highlights the social influences on environmental health.<br>The accelerated urbanisation of Morocco, expansion in vehicle numbers, and infrastructural promotion add to the aggravation of noise pollution, as is the case with other developing states.<br>Nevertheless, in contrast to some European states possessing fixed systems of noise management and monitoring (like the Environmental Noise Directive of the EU), Morocco has no well-developed noise maps, data frameworks, and integrated involvement of stakeholders in noise policy.<br>Lack of specific clinical and epidemiological investigations that associate the noise exposure with the health outcomes in Morocco poses a major constraint, hence lesser levels of evidence are sure.<br>Such a ToA difference is reflective of the overlaying regional and more global evidence base, which, according to longitudinal and physiological research, supports causal conclusions.<br>These results indicate that Morocco will be in a position to enjoy internationally recognised best practices, especially those of the European Union, but adapted to local socioeconomic and urban conditions.<br>Some of the recommendations have focused on making noise limit enforcement, creating noise mapping programmes nationally, ensuring that noise control is incorporated in city planning, as well as enhancing public awareness.<br>The review highlights the role of multi-sectoral partnership in which policy-makers, city planners, and governments in developing communities should work together to enable the development of effective, localized noise management strategies.<br>Here, in principle, the review contextualizes Moroccan noise pollution issues to a global discourse, pointing to common challenges in urban noise control combined with specific shortcomings in terms of legislation, implementation, data acquisition, and health studies.<br>It supports the idea that this will help Morocco to use regional and international examples, technical aid, and community engagement to develop more robust noise control policies that safeguard the health of people and reduce environmental inequalities.<br>This presentation offers a moderate, evidence-based viewpoint that captures the existing state of knowledge as well as enables future developments, which makes this review much more relevant and practical in its effects on policy.	
	23b	Several limitations that impact the strength and applicability of the findings:<br>• Small Size and Scope: There are only 23 Morocco-specific investigations in the corpus, only partly materialized by more general and even international bibliography, so it represents a lack of research on the issue of noise pollution, its health effects, and its regulatory effectiveness in the Moroccan context.<br>• Study Heterogeneity: The sampled studies have significant differences in design, methodology, noise-measurement methods, and findings, which present a significant barrier to comparing and synthesizing results.<br>• Data Quality and Completeness Many studies lack noise-exposure, standardized measurement, or comprehensive studies on testing health outcomes; in addition, many of these datasets are outdated or geographically limited.	-

Section and Topic	Item #	Checklist item	Location where item is reported
		• Lack of Clinical and Epidemiological Research: There is a glaring gap in direct clinical findings and longitudinal investigations, which makes it impossible to provide unquestionable evidence on mediating noise exposure and certain health outcomes in the context of Moroccan populations and consequently mitigate causal conclusions.<br>• Regulatory Voids and Perpetual Vices: Although the rules are in place in Morocco to create the legal basis of noise-pollution laws, these laws are not enforced rigorously, there are no strictly defined permissible noise limits, and there is no systematic noise mapping, noise monitoring frameworks, which limits the evidence of practical noise control.<br>• Limited Public and Stakeholder Consultations: There is a lack of public consciousness, minimal communal and multisector stakeholder viewpoints, and thought of the noise policy development, leading to a decrease in data richness and policy pertinence.<br>• Absence of Meta-Analysis and Quantitative Synthesis Breadth The hostile heterogeneity of studies and lack of numeric data bar any formal statistical synthesis/meta-analysis, to such a degree that it restricts the offering of accurate effect estimates and quantitative evaluation of heterogeneity.<br>• Reporting and Publication Biases: There is a lack of official reporting and publication bias evaluation related to undocumented missing results or selective discussion of results to a specific topic, or being selective due to the limitation of regional research funds.	
	23c	The review methods that were utilized during the systematic review on noise pollution in Morocco are thus not without limitations and can be summarized as follows:<br>• Major mainly qualitative synthesis: The review mainly bases itself on qualitative analysis of narratives because of the apparent heterogeneity of the studies considered and scarcity of persistent quantitative measures, which limits the possibility of meta-analyses or strong synthesis in terms of statistics.<br>• Heterogeneous Study Designs: Studies included vary massively in their methods, outcomes, and noise measurements, and pooling analysis and interpreting them comparatively are difficult.<br>• Formal risk of bias assessment methods (e.g., RoB 2, ROBINS-I, or risk of bias tables for individual studies) were not used, which limits transparency regarding study quality, despite data extraction by several independent reviewers.<br>• No formal testing of reporting bias: No formal test of publication or reporting bias is conducted in the review, e.g., of funnel plots or selective outcome reporting tests.<br>• Limited sensitivity analyses: No sensitivity analyses were conducted to determine how strong findings could be in terms of including the study in the study or the methodology.<br>• Limits potential literature search limitations, although various databases and official sources were examined systematically, only English age or French may not retrieve the relevant study, excluding publications not in English or French (in addition to key data). Grey literature or unpublished data can also be under-represented, which can also lead to publication bias.<br>• Data extraction constraints: The unstructured nature of major study properties and findings prevents a more straightforward display, and some of the data impacting the research might not be displayed in a consistent fashion among studies.<br>• Sparsity of the high-quality data: There is a limited amount of available data from high-quality epidemiological and longitudinal studies to enhance the strength of evidence synthesis that is possible due to the review process.<br>• Benign involvement of stakeholders or communities: The review does not mention any forward postulation concerning any stakeholders or communities involved in the refinement of research questions and interpretation of the findings, which would make them more meaningful and contextually relevant.	-

Section and Topic	Item #	Checklist item	Location where item is reported
		These limitations are part of the nature of the subject matter, as well as the limitations of the evidence base are relevant. A number of these concerns are acknowledged in the review; updates in the future may be enhanced by integrating formal risk of bias evaluations, extending inclusion operated as well as by realizing a quantitative synthesis where practicable, as well as by involving pertinent stakeholders in the whole review method.	
	23d	The results of this systematic review have important implications for practice, policy, and future research related to noise pollution in Morocco:<br>Practice:<br>• Urban planners, the social and environmental health community should take noise pollution as a serious issue, and the issue of noise management should be incorporated into the urban development and health promotion programmes.<br>• Some grins are required that an improved noise monitoring scheme be put in place in large cities in Morocco to monitor exposure levels, record hotspots, and communicate them to targeted curbing schemes, e.g., noise blockers, traffic restraint, zoning, etc.<br>• The field of vulnerable populations in low-income neighbourhoods needs specific interventions to mitigate the high noise exposures and the related health inequities.<br>• General healthcare environments must consider the issue of occupational noise exposure as a stressor and cause of burnout among workers and introduce noise control and acuity measures.<br>Policy:<br>• Morocco should formulate an integrated national policy on noise-reduction policies that include enforceable noise limits based on areas (residential, commercial, industrial) and time of the day to meet the WHO and international standards.<br>• It is essential to institute a national noise-mapping programme so that evidence-based regulation, enforcement, and urban planning can take place.<br>• Improvement of the enforcement procedures and fines when notification is breached, coupled with capacity building of environmental agencies, will assist in compliance.<br>• Policymakers need to promote the collaboration of multiple sectors (including urban planners, health authorities, environmental agencies, and civil society) to implement noise management frameworks that are integrated.<br>• International best practices, including the Environmental Noise Directive of the European Union, and adapting it to the Moroccan societal and economic environment, are a means through which improved regulatory efficiency can be achieved.<br>Future Research:<br>• The research gap on direct clinical and epidemiological evidence linking noise exposure to health outcomes in Morocco must be addressed with longitudinal cohort and physiological studies.<br>• Standardized and harmonized noise exposure assessment methods should be adopted to improve data quality and comparability across studies.<br>• Investigations into the effectiveness of specific noise mitigation interventions in Moroccan urban settings are warranted.<br>• Socioeconomic and environmental justice dimensions of noise pollution deserve further exploration to inform equitable policy actions.<br>• Development of centralized, accessible noise data repositories will support ongoing monitoring, research, and policy evaluation.	

Section and Topic	Item #	Checklist item	Location where item is reported
		Overall, the review highlights the necessity of Morocco to expand the current legal frameworks of noise by implementing effective, enforceable noise laws, increasing surveillance and research abilities, and stakeholder involvement across different sectors. This will increase protection of public health and help create more sustainable and healthy urban environments with the ongoing city-urbanisation and urbanisation.	
OTHER INFORMATION			
Registration and protocol	24a	The review was not registered.	-
	24b	The review protocol was not prepared for this systematic review.	-
	24c	No amendments are provided.	-
Support	25	No specific financial support was applied for or received to conduct this systematic review	-
Competing interests	26	The authors of the review declare that there are no competing interests disclosed that might affect the objectivity or integrity of the review.	Page 24
Availability of data, code and other materials	27	No additional materials are publicly available.	-

*This checklist is reproduced and adapted from Page MJ, McKenzie JE, Bossuyt PM, Boutron I, Hoffmann TC, Mulrow CD, et al. The PRISMA 2020 statement: an updated guideline for reporting systematic reviews. BMJ 2021;372:n71. doi: 10.1136/bmj.n71. This work is licensed under CC BY 4.0. To view a copy of this license, visit https://creativecommons.org/licenses/by/4.0/.
